# Evaluation of the Efficacy of ChAd63-MVA Vectored Vaccines Expressing Circumsporozoite Protein and ME-TRAP Against Controlled Human Malaria Infection in Malaria-Naive Individuals

**DOI:** 10.1093/infdis/jiu579

**Published:** 2014-10-21

**Authors:** Susanne H. Hodgson, Katie J. Ewer, Carly M. Bliss, Nick J. Edwards, Thomas Rampling, Nicholas A. Anagnostou, Eoghan de Barra, Tom Havelock, Georgina Bowyer, Ian D. Poulton, Simone de Cassan, Rhea Longley, Joseph J. Illingworth, Alexander D. Douglas, Pooja B. Mange, Katharine A. Collins, Rachel Roberts, Stephen Gerry, Eleanor Berrie, Sarah Moyle, Stefano Colloca, Riccardo Cortese, Robert E. Sinden, Sarah C. Gilbert, Philip Bejon, Alison M. Lawrie, Alfredo Nicosia, Saul N. Faust, Adrian V. S. Hill

**Affiliations:** 1 Jenner Institute; 2 Centre for Statistics in Medicine; 3 Clinical Biomanufacturing Facility, University of Oxford; 4 NIHR Wellcome Trust Clinical Research Facility, University of Southampton and University Hospital Southampton NHS Foundation Trust; 5 Division of Cell and Molecular Biology, Imperial College London, United Kingdom; 6 Royal College of Surgeons in Ireland, Dublin, Ireland; 7 Okairos, Rome; 8 CEINGE; 9 Department of Molecular Medicine and Medical Biotechnology, University of Naples Federico II, Italy; 10 Okairos, Basel, Switzerland; 11 Centre for Geographical Medical Research (Coast), Kenya Medical Research Institute–Wellcome Trust,Kilifi

**Keywords:** malaria, *P. falciparum*, vaccine, ChAd63, MVA, ME-TRAP, CS, CHMI

## Abstract

***Background.*** Circumsporozoite protein (CS) is the antigenic target for RTS,S, the most advanced malaria vaccine to date. Heterologous prime-boost with the viral vectors simian adenovirus 63 (ChAd63)-modified vaccinia virus Ankara (MVA) is the most potent inducer of T-cells in humans, demonstrating significant efficacy when expressing the preerythrocytic antigen insert multiple epitope–thrombospondin-related adhesion protein (ME-TRAP). We hypothesized that ChAd63-MVA containing CS may result in a significant clinical protective efficacy.

***Methods.*** We conducted an open-label, 2-site, partially randomized *Plasmodium falciparum* sporozoite controlled human malaria infection (CHMI) study to compare the clinical efficacy of ChAd63-MVA CS with ChAd63-MVA ME-TRAP.

***Results.*** One of 15 vaccinees (7%) receiving ChAd63-MVA CS and 2 of 15 (13%) receiving ChAd63-MVA ME-TRAP achieved sterile protection after CHMI. Three of 15 vaccinees (20%) receiving ChAd63-MVA CS and 5 of 15 (33%) receiving ChAd63-MVA ME-TRAP demonstrated a delay in time to treatment, compared with unvaccinated controls. In quantitative polymerase chain reaction analyses, ChAd63-MVA CS was estimated to reduce the liver parasite burden by 69%–79%, compared with 79%–84% for ChAd63-MVA ME-TRAP.

***Conclusions.*** ChAd63-MVA CS does reduce the liver parasite burden, but ChAd63-MVA ME-TRAP remains the most promising antigenic insert for a vectored liver-stage vaccine. Detailed analyses of parasite kinetics may allow detection of smaller but biologically important differences in vaccine efficacy that can influence future vaccine development.

***Clinical Trials Registration.*** NCT01623557.

The worldwide burden of *P. falciparum* malaria remains a major public health concern [1], with approximately 207 million cases and 627 000 deaths worldwide in 2012 [2]. The preerythrocytic *P. falciparum* vaccine RTS,S, formed from fusion of the circumsporozoite protein (CS) to the surface-antigen of hepatitis B virus, is the most advanced malaria vaccine in development. However, it confers only limited, relatively short-lived protection in African infants [3–5]. Analysis of the immunological correlates of immunity induced by RTS,S suggests that high levels of antibodies against CS on the sporozoite correlate with protection, with a possible minor contribution from low levels of induced CD4^+^ T cells [6–8]. While these clinical results are the most effective to date in a field setting, there remains a need to improve on this limited clinical efficacy [9, 10], either through modifications to RTS,S or by developing vaccine strategies that combine numerous antigens or vaccine platforms.

Increasingly, data from animal models and vectored immunizations demonstrate a correlation between CD8^+^ T cells and immunity to liver-stage parasites, even in the absence of antibodies [11–17]. Clinical vaccine development had been hampered by the limited ability of traditional subunit vaccine strategies, namely adjuvanted protein constructs, to induce high enough numbers of antigen-specific CD8^+^ T cells that may confer protection [18]. However, more recently, adenoviral-vectored malaria vaccines administered in heterologous prime-boost regimens with a modified vaccinia virus Ankara (MVA) boost have been capable of inducing good humoral and T-cell responses that include high levels of CD8^+^ T cells [17–21]. These CD8^+^ T-cell responses have been associated with clinical efficacy [17]. Given concerns regarding the effect of preexisting immunity on the immunological potency of human adenoviruses, simian adenoviruses (ChAd) are being developed as alternative, potent vectors [22]. Indeed, prime-boost vaccination with ChAd63 and MVA expressing the leading preerythrocytic antigen, ME-TRAP, is clinically the most potent inducer of CD8^+^ T cells in humans and the most effective malaria vaccine besides RTS,S, demonstrating efficacy, defined as sterile protection or delay, in 8 of 14 malaria-naive volunteers (57%) following sporozoite challenge [17].

Given that CS is expressed during both the sporozoite and liver stages of *P. falciparum* infection and therefore is possibly susceptible to both humoral and cell-mediated immunity at both stages, we assess here the efficacy of ChAd63-MVA expressing CS. If effective, this vaccine could then be combined with ChAd63-MVA expressing ME-TRAP or RTS,S, to improve clinical efficacy. Following a phase 1a study of ChAd63-MVA CS in malaria-naive volunteers, in which the regimen was shown to be safe and immunogenic (de Barra et al, submitted), we performed a study of controlled human infection with *Plasmodium* sporozoites (also known as “controlled human malaria infection” [CHMI]) [23], using the standard challenge model involving infectious bites from 5 mosquitoes, to compare the efficacy of ChAd63-MVA CS with that of ChAd63-MVA ME-TRAP.

## METHODS

### Participants

The study was conducted at the Centre for Clinical Vaccinology and Tropical Medicine, University of Oxford (Oxford, United Kingdom), and at the National Institute for Health Research (NIHR) Wellcome Trust Clinical Research Facility, part of the University of Southampton and University Hospital Southampton National Health Service (NHS) Foundation Trust (Southampton, United Kingdom). The challenge procedure was performed as previously described [24], using 5 infectious bites from *P. falciparum* strain 3D7–infected *Anopheles stephensi* mosquitoes. This took place at the Alexander Fleming Building, Imperial College (London, United Kingdom), and mosquitoes were supplied by the Department of Entomology, Walter Reed Army Institute of Research (WRAIR; Washington, DC). Healthy, malaria-naive men and non-pregnant women aged 18–45 years were invited to participate in the study. All volunteers gave written informed consent prior to participation, and the study was conducted according to the principles of the Declaration of Helsinki and in accordance with good clinical practice. There was no selection of volunteers on the basis of preexisting neutralizing antibodies to the ChAd63 vector before enrollment. The full list of inclusion and exclusion criteria is given in the Supplementary Materials.

### Ethical and Regulatory Approval

All necessary approvals for the study were granted by the United Kingdom National Research Ethics Service, Committee South Central–Oxford A (reference 12/SC/0037), and the United Kingdom Medicines and Healthcare Products Regulatory Agency (reference 21584/0293/001-0001). The study was additionally reviewed by the Western Institution Review Board (Seattle, WA; reference 20120266) at the request of the PATH Malaria Vaccine Initiative and was approved. The Genetically Modified Organisms Safety Committee of the Oxford University Hospitals NHS Trust (reference GM462.11.65) authorized recombinant vaccine use. The trial was registered with ClinicalTrials.gov (reference NCT01623557). The local safety committee provided safety oversight, and good clinical practice compliance was independently monitored by an external organization (Appledown Clinical Research, Great Missenden, United Kingdom).

### ChAd63 and MVA Vaccines

Generation, manufacture, and quality control monitoring of the recombinant ChAd63 and MVA vectors encoding ME-TRAP and CS have been previously described [de Barra et al, submitted; 25]. The antigen ME-TRAP contains a fusion protein of a multi-epitope string (ME), followed by preerythrocytic thrombospondin-related adhesion protein (TRAP) from *P. falciparum* strain T9/96 [17].

The poor immunogenicity of the standard full-length CS insert (CSO) previously used in clinical trials by our group [26–29] suggested that there may be an important difference in the intrinsic immunogenicity of CSO, compared with that of the ME-TRAP insert. For this study, we used information from multiple sources [30–32] to design a novel CS antigen that omits the extreme C-terminus of the protein that encodes the glycophosphatidylinositol anchor sequence and may down-modulate CS immunogenicity [de Barra et al, submitted; 33].

### Study Design

**Figure 1.**
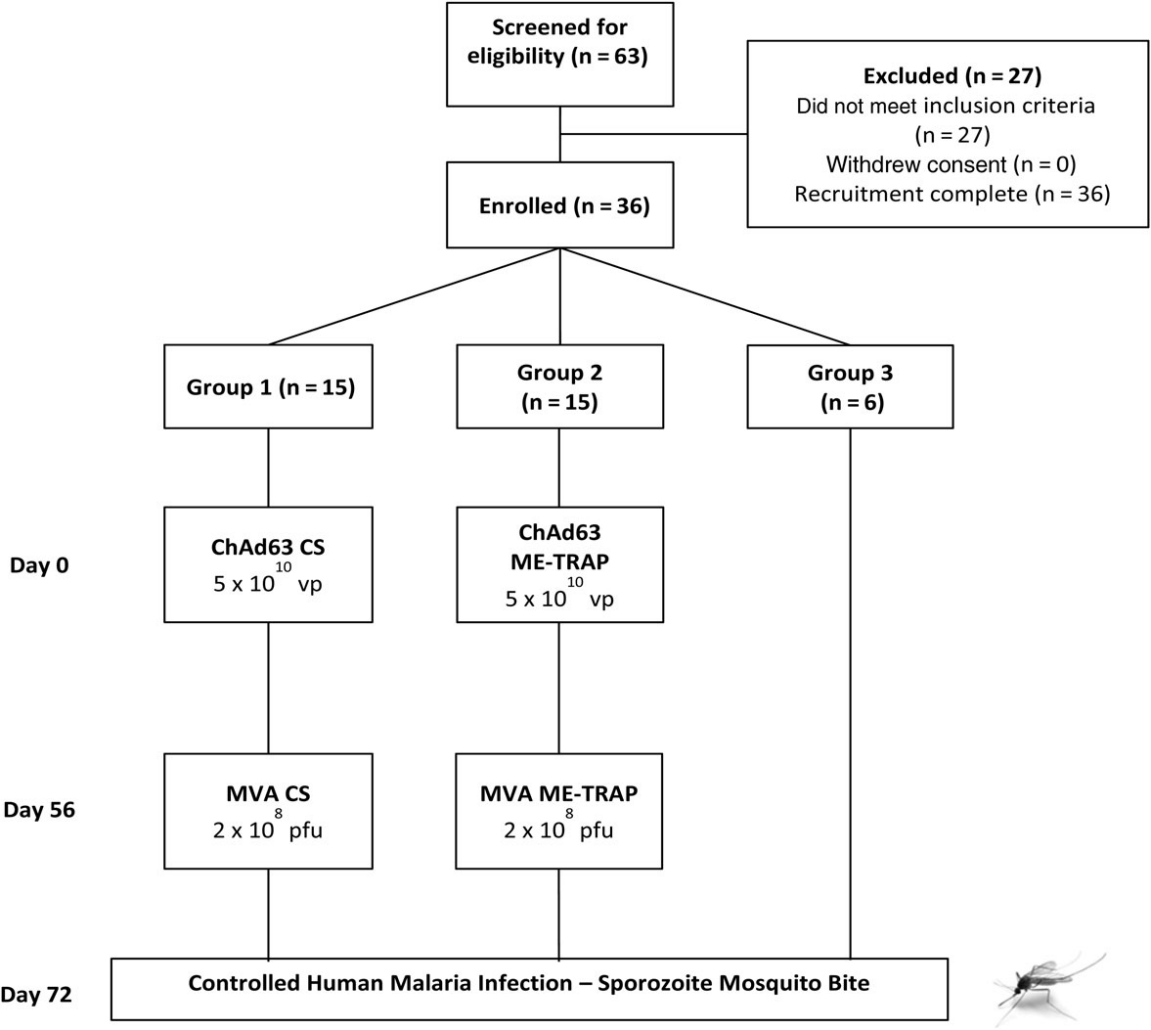
Flow of study design and volunteer recruitment. Twenty volunteers were excluded following screening for the following reasons: psychiatric history (n = 3), no medical screening letter returned (n = 3), multiple medical problems (n = 2), excessive alcohol use (n = 2), syncope (n = 1), connective tissue disease (n = 1), iron deficiency (n = 1), raised alanine aminotransferase level (n = 1), poor venous access (n = 1), gastrointestinal problems under investigation (n = 1), family history of heart disease (n = 1), lost to follow-up (n = 1), unavailable during challenge (n = 1), and history of recreational drug use (n = 1). Furthermore, 7 volunteers withdrew consent after screening but before enrollment. All immunizations were administered intramuscularly with sequential vaccines administered into the deltoid of alternating arms. No enrolled volunteers withdrew from the study and all volunteers completed study visits as scheduled. Abbreviations: ChAd63, simian adenovirus 63; CS, circumsporozoite protein; ME-TRAP, multiple epitope–thrombospondin-related adhesion protein; MVA, modified vaccinia virus Ankara; pfu, plaque-forming units; vp, viral particles.

This was a Phase I/IIa open-label, vaccine and CHMI trial (Figure 1). Volunteers chose whether to participate as vaccinees (groups 1 and 2) or unvaccinated controls undergoing CHMI alone (group 3). Vaccinees were randomly allocated to groups 1 or 2. All vaccinations were administered intramuscularly into the deltoid, with the ChAd63 and MVA-vectored vaccines administered in alternating arms. ChAd63-vectored vaccines were administered on day 0, and MVA boost was administered on day 56. Details of dosing, clinical follow-up and safety monitoring are given in Supplementary Information. An interval of 1–14 days was allowed between vaccination and follow-up visits after vaccination. CHMI was performed on day 77. Throughout this article, “study day” refers to the nominal time point for a group and not the actual day of sampling.

### Ex Vivo Interferon γ (IFN-γ) Enzyme-Linked Immunosorbent Spot (ELISPOT) Analysis

Ex vivo (18-hour stimulation) ELISPOT assays for ME-TRAP and CS were performed on fresh (ie, not previously frozen) peripheral blood mononuclear cells (PBMCs) from blood samples obtained on days 0, 14, 28, 56, and 63 after vaccination and on 1 day before and 7, 35, and 90 days after CHMI. Antigens were tested in duplicate with 250 000 freshly isolated PBMCs added to each well. Details about the ELISPOT methods are available in the Supplementary Materials.

### Total Immunoglobulin G (IgG) Enzyme-Linked Immunosorbent Assay (ELISA)

Antibody responses were assessed using serum samples collected on days 0, 28, 56, and 63 after vaccination and 1 day before and 35 and 90 days after CHMI. Antibody responses to TRAP were measured by an IgG ELISA performed at the Jenner Institute (Oxford; Supplementary Materials). Antibody responses to CS were measured by an IgG ELISA performed at the WRAIR International Reference Center for Malaria Serology (Supplementary Materials) [34].

### Parasite Quantitative Polymerase Chain Reaction (qPCR)

qPCR for *P. falciparum* was conducted as described previously [35] (see Supplementary Materials).

### Criteria for Malaria Diagnosis

Diagnosis of malaria following CHMI was defined as positive findings of thick film microscopy, with at least 1 morphologically normal malaria trophozoite seen by ≥1 experienced microscopist. qPCR was simultaneously performed, although investigators directly involved in clinical management were blinded to these results. For volunteers with positive findings of thick film microscopy but no symptoms consistent with *P. falciparum* infection, investigators were unblinded to the qPCR results, with the volunteer treated only if any preceding samples had >500 parasites/mL. For volunteers with symptoms or signs that, in the opinion of the clinical investigators, likely represented malaria (eg, fever, rigors, or severe symptomatology), despite negative findings of thick film microscopy and no alternative cause, investigators were unblinded to the qPCR results. If any volunteer's preceding samples had >500 parasites/mL, the volunteer was treated for malaria. A vaccinee was classified as a participant who demonstrated a delay to patency/treatment if treatment was started >2 times the standard deviation in days after the mean time to treatment of unvaccinated control volunteers. This corresponds to clearance of an estimated >95% of preerythrocytic-stage parasites [36].

### Statistical Analysis

Data were analyzed using GraphPad Prism, version 5.03 for Windows (GraphPad Software, La Jolla, California). Individual, geometric mean (GM), or median responses for measurements within each group are described. Parasite densities were log transformed to remove skewness, with 1 added to each value to allow transformation of zero values. Significance testing of differences between groups used either a 2-tailed *t* test or the 2-tailed Mann–Whitney test (or the Kruskal–Wallis test, for comparisons of >2 groups) for nonparametrically distributed data. Correlations were assessed using the Spearman rank correlation coefficient. Time to treatment was analyzed using Kaplan–Meier survival curves, and between-group comparisons were made using the log-rank test.

## RESULTS

### Recruitment and Vaccinations

Recruitment took place between March and June 2012. Thirty healthy malaria-naive adult volunteers (10 women and 20 men) were enrolled as vaccinees across 2 sites in the United Kingdom. Six further volunteers (5 women and 1 man) were enrolled to undergo CHMI as unvaccinated infectivity controls (Figure 1). The mean age of volunteers was 26.4 years (range, 19–40 years). Vaccinations began in April 2012, CHMI occurred in July 2012, and all follow-up visits were completed by November 2012. All vaccinees received their immunizations as scheduled. All doses of vaccines were the same as those used in the comparable phase 1a studies [de Barra et al, submitted; 25]. All volunteers underwent CHMI 15–21 days after MVA immunization (ie, on days 71–77).

### Vaccine Safety and Reactogenicity

No unexpected or serious adverse events (AEs) related to vaccination occurred. The local and systemic (Supplementary Figure 1) reactogenicity profile of each vaccine was similar to phase 1a data [de Barra et al, submitted; 25].

### T-Cell Immunogenicity to ChAd63-MVA CS and ME-TRAP

**Figure 2.**
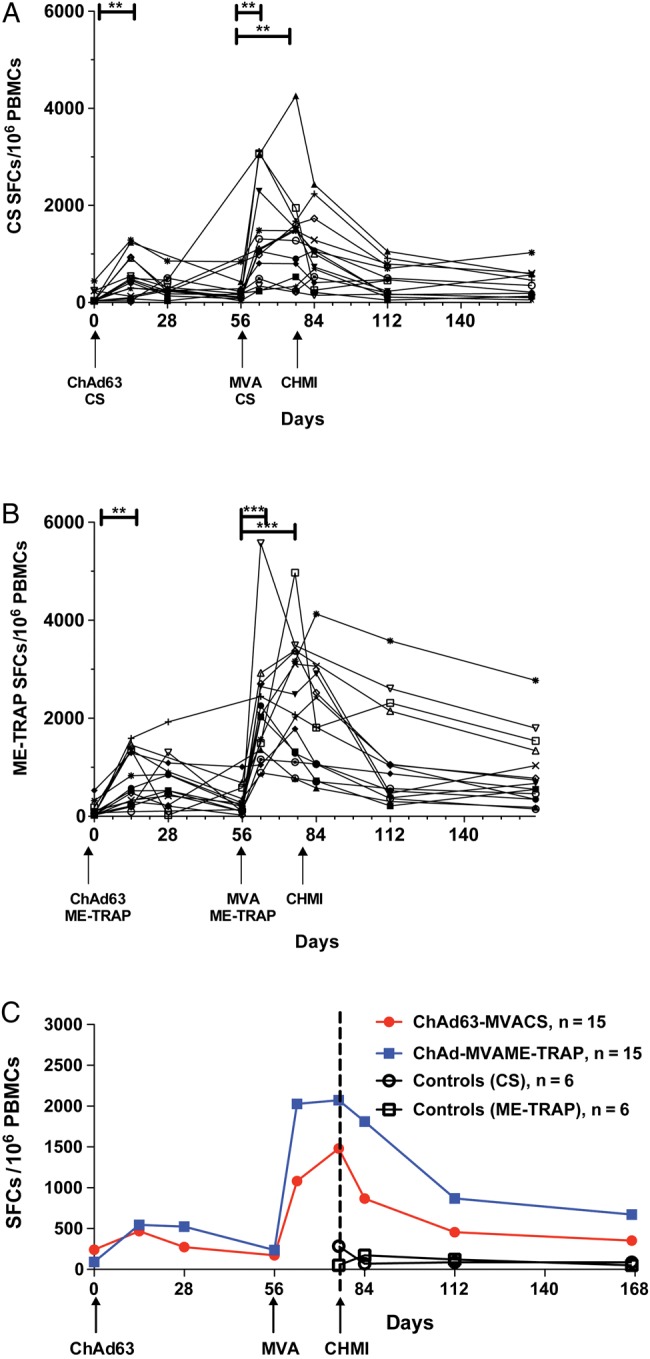
Antigen-specific T-cell responses to vaccination measured by interferon γ enzyme-linked immunosorbent spot assay. Kinetics of T-cell responses after vaccination with ChAd63-MVA encoding either circumsporozoite protein (CS; group 1; *A*) or ME-TRAP (group 2; *B*). Each line represents an individual volunteer. **P < .01 and ***P < .001, by the Kruskal–Wallis test with the Dunn multiple comparison test. *C*, Median T-cell frequencies for both antigens by group. Mean T-cell frequencies at day 28 after vaccination were 304 and 673 spot-forming cells (SFCs) after ChAd63-MVA CS or ME-TRAP receipt, respectively, and at day 63 peaked at 1378 and 2068 SFCs after ChAd63-MVA CS or ME-TRAP receipt, respectively. Abbreviations: ChAd63, simian adenovirus 63; CHMI, controlled human malaria infection; controls, unvaccinated volunteers undergoing CHMI; ME-TRAP, multiple epitope–thrombospondin-related adhesion protein; MVA, modified vaccinia virus Ankara; PBMC, peripheral blood mononuclear cell.

T-cell responses followed the expected kinetics after ChAd63 receipt [de Barra et al, submitted; 17, 25, 35, 37, 38], with peak responses seen 28 days after ChAd63 receipt (group 1 [CS]: GM, 343 spot-forming cells (SFCs)/million PBMCs [95% CI, 191–617]; group 2 [ME-TRAP]: GM, 553 SFCs/million PBMCs [95% CI, 330–925]). The peak T-cell response after boost was seen at day 63 after receipt of MVA CS for group 1 (GM, 1017 SFCs/million PBMCs [95% CI, 630–1641]) and at 1 day before CHMI after MVA ME-TRAP receipt for group 2 (GM, 2027 SFCs/million PBMCs [95% CI, 1472–2792]; Figure 2*A* and 2*B*). There was no significant difference in T-cell responses between day 63 after vaccination and 1 day before CHMI for either group.

Responses to both antigens were well maintained, with GMs of 285 SFCs/million PBMCs (95% CI, 156–520) to CS and 659 SFCs/million PBMCs (95% CI, 418–1036) to ME-TRAP 16 weeks after MVA receipt in groups 1 and 2, respectively (Figure 2*C*). T-cell responses among infectivity controls showed a GM of 110 SFCs/million PBMCs (95% CI, 40–304) to CS and a GM of 85 SFCs/million PBMCs (95% CI, 31–231) to ME-TRAP 1 day before CHMI. These responses did not change significantly during follow-up (Figure 2*C*).

Detailed mapping of T-cell responses to the ME-TRAP antigen are outlined in the Supplementary Materials. Detailed mapping of T-cell responses to CS peptides was not performed because this was described recently in detail with several HLA class I–restricted epitopes [39].

### Antibody Immunogenicity of ChAd63-MVA CS and ME-TRAP

**Figure 3.**
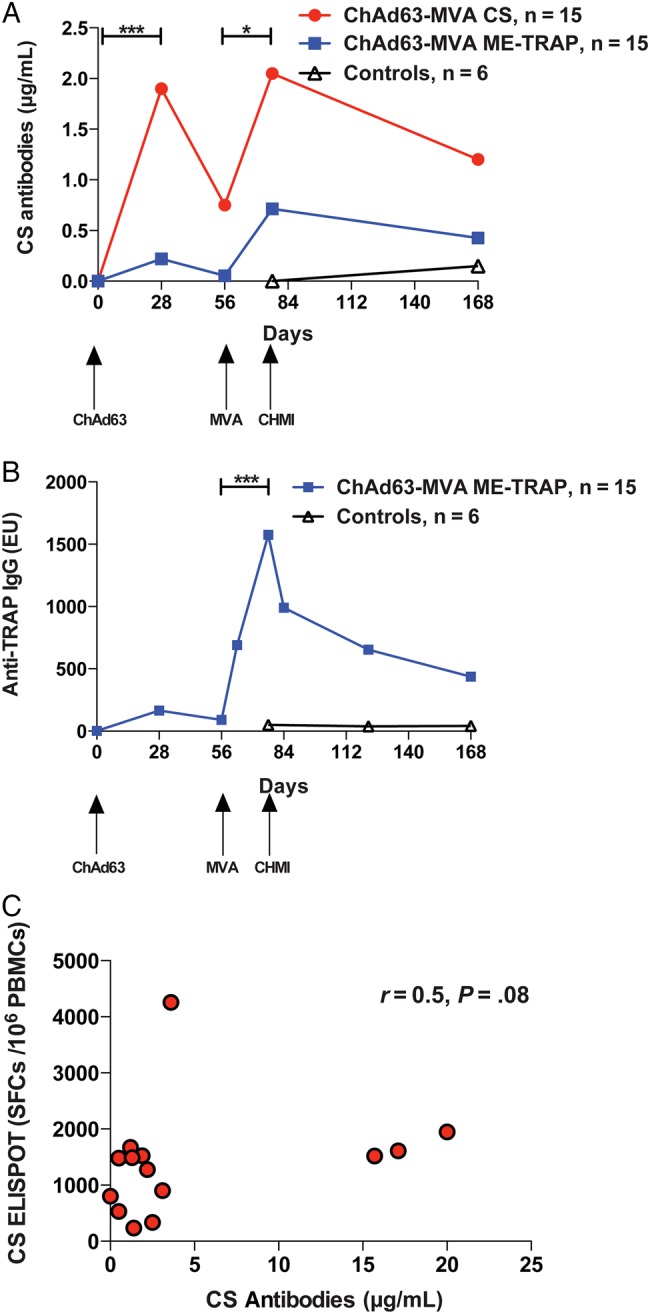
Antibody responses to vaccination measured by enzyme-linked immunosorbent assay (ELISA). *A*, Anti- circumsporozoite protein (CS) immunoglobulin G (IgG) antibody responses after vaccination with ChAd63-MVA CS (group 1; red) or ME-TRAP (group 2; blue). Lines represent group medians. ***P = <.001 and *P = <.05, by the Friedman test comparing responses before and after vaccination with the Dunn post hoc test. *B*, Anti-TRAP IgG antibody responses after vaccination with ChAd63 ME-TRAP (group 2). ***P = .0002, by the 2-tailed Wilcoxon matched pairs test. *C*, Correlation between anti-CS IgG antibodies and CS-specific T-cell immunogenicity the day before challenge in group 1. Spearman *r* = 0.5; *P* = .08*.* Abbreviations: ChAd63, simian adenovirus 63; CHMI, controlled human malaria infection; controls, unvaccinated volunteers undergoing CHMI; ELISPOT, enzyme-linked immunosorbent spot assay; EU, ELISA units; ME-TRAP, multiple epitope–thrombospondin-related adhesion protein; MVA, modified vaccinia virus Ankara; PBMC, peripheral blood mononuclear cell; SFC, spot-forming cell.

Anti-CS IgG antibody responses were measured in all vaccinees (Figure 3*A*). Anti-CS IgG antibodies were detected in ME-TRAP vaccinees (group 2) because of the inclusion of 4 copies of the N-acetylneuraminic acid phosphatase (NANP) repeat from the CS antigen in the ME string. In group 1, anti-CS IgG responses peaked 21 days after MVA receipt, with a median level of 2.1 µg/mL. In group 2, anti-CS IgG responses also peaked 21 days after MVA, but 8 of 14 volunteers in this group did not have a measurable response, giving a median level of 0 µg/mL. Anti-TRAP IgG antibody responses were assessed in group 2 only (Figure 3*B*) and also peaked 21 days after MVA ME-TRAP receipt (median, 1475 ELISA units). A weak relationship between anti-CS IgG antibody responses and CS-specific T-cell responses 1 day before CHMI was observed in group 1 (*r* = 0.5; *P* = .08, by 2-tailed Spearman correlation; Figure 3*C*). Exposure to CHMI did not induce significant levels of anti-CS or TRAP antibodies among infectivity controls (Figure 3*A* and 3*B*).

### ChAd63-MVA Efficacy Among All Regimens Following Sporozoite Challenge

**Figure 4.**
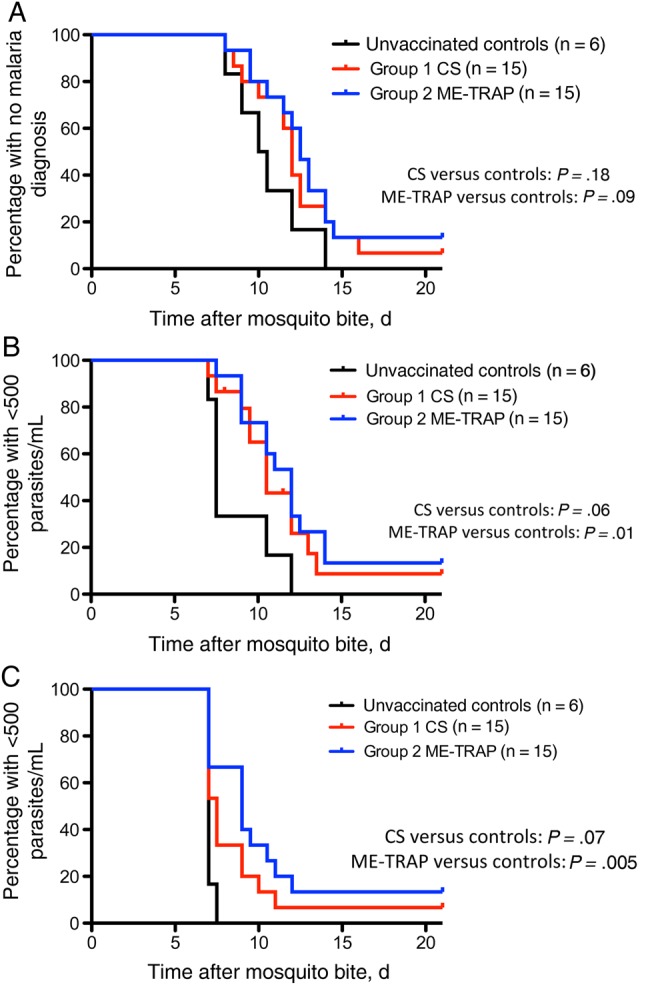
Efficacy of ChAd63-MVA circumsporozoite protein (CS) and ME-TRAP immunization following *Plasmodium falciparum* 3D7 sporozoite challenge. Kaplan–Meier survival analyses. Log-rank test for significance. *A*, Kaplan–Meier survival analysis of time to treatment. Median time, 12.0 days for group 1 (CS), 12.5 days for group 2 (ME-TRAP), and 10.3 days for unvaccinated controls. *B*, Kaplan–Meier survival analysis of time to first sample with >500 parasites/mL detected by quantitative polymerase chain reaction (qPCR). Median time, 10.5 days for group 1 (CS), 12.0 days for group 2 (ME-TRAP), and 7.5 days for unvaccinated controls. *C*, Kaplan–Meier survival analysis of time to first sample with >20 parasites/mL detected by qPCR. Median time, 7.5 days for group 1 (CS), 9.0 days for group 2 (ME-TRAP), and 7.0 days for unvaccinated controls. Abbreviations: CHMI, controlled human malaria infection; controls, unvaccinated volunteers undergoing CHMI; ME-TRAP, multiple epitope–thrombospondin related adhesion protein.

The infectivity controls (group 3) and 27 of 30 vaccinees were diagnosed with malaria. One volunteer (7%) in group 1 (who received ChAd63-MVA CS) and 2 volunteers (13%) in group 2 (who received ChAd63-MVA ME-TRAP) were sterilely protected (Figure 4*A*). The control volunteers (group 3) were diagnosed after a median time of 10.3 days, mean time of 10.5 days (range 8.0--14.0, SD 2.2). Three vaccinees (20%) in group 1 and 5 vaccinees (33%) in group 2 demonstrated a delay in time to treatment, relative to controls. There was no significant difference between unvaccinated controls and vaccinees in the protocol-specified end point of time to treatment for malaria (Figure 4*A*). However, when comparing the time to collection of the first sample after CHMI with either >500 parasites/mL (Figure 4*B*) or >20 parasites/mL (Figure 4*C*), a significant difference was seen between unvaccinated controls and vaccinees receiving ChAd63-MVA ME-TRAP (*P* = .01 and *P* = .005, respectively).

### qPCR Data

**Figure 5.**
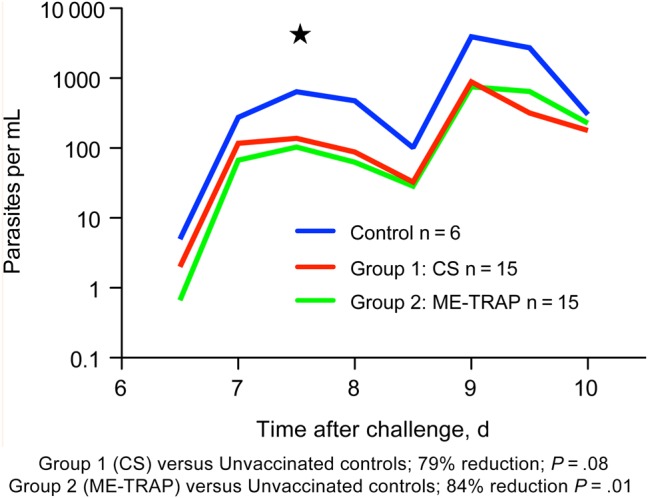
Comparison of mean parasite density, measured by quantitative polymerase chain reaction, 7.5 days after controlled human malaria infection (CHMI) between vaccinees and control volunteers. *P* values were determined by the Mann–Whitney *U* test*.* Abbreviations: ChAd63, simian adenovirus 63; Control, unvaccinated volunteers undergoing CHMI; CS, circumsporozoite protein; group 1, ChAd63-MVA CS recipients; group 2, ChAd63 ME-TRAP recipients; ME-TRAP, multiple epitope–thrombospondin-related adhesion protein; MVA, modified vaccinia virus Ankara.

Primary analysis comparing the mean parasite density 7.5 days after CHMI (a measure of the liver to blood inoculum) showed a significant reduction when vaccinees receiving ChAd63-MVA ME-TRAP but not ChAd63-MVA CS were compared with unvaccinated control volunteers (*P* = .01 and *P* = .08, respectively, by the Mann–Whitney *U* test; Figure 5). The same comparison performed using negative binomial regression gave *P* values of .03 and .05, and a similar result was seen when the liver to blood inoculum was estimated 7.5 days after CHMI by using simple linear regression (*P* = .01 and *P* = .05, by the Mann–Whitney *U* test)*.* Mean total number of parasites 7.5 days after CHMI was a strong predictor of the time to treatment (hazard ratio [HR], 1.003974 [95% CI, 1.002272–1.00568], by Cox proportional hazards regression analysis; *P* ≤ .0001).

**Figure 6.**
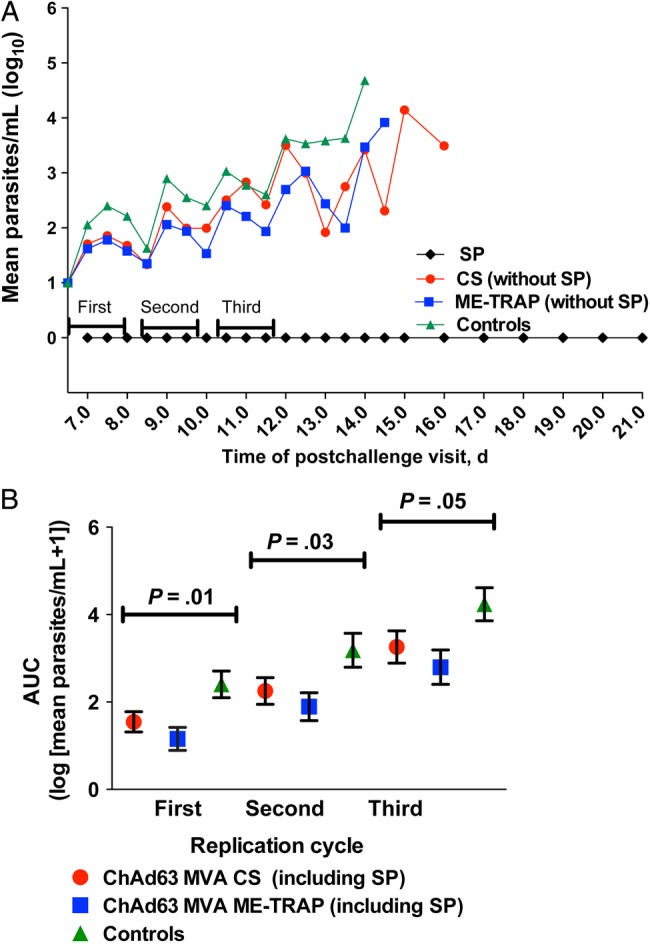
Comparison of areas under the curve (AUCs) of parasite densities, measured by quantitative polymerase chain reaction (PCR), between vaccinees and control volunteers. *A*, Group mean log-transformed PCR data. The AUC of parasite density over the ﬁrst 3 replication cycles in infected volunteers was a signiﬁcant predictor of the time to diagnosis (hazard ratio, 1.000015 [95% confidence interval, 1.000008–1.000022], by Cox proportional hazards regression analysis; *P* < .000). *B*, AUC analysis of parasite densities, comparing controls to vaccinees at days 6.5–8 (the first cycle after hepatocyte release), days 8.5–10 (the second cycle), and days 10.5–12 (the third cycle) after controlled human malaria infection (CHMI). Means of log [parasite density + 1] were compared for each vaccine group to those of controls, using a 2-tailed *t* test. Abbreviations: ChAd63, simian adenovirus 63; controls, unvaccinated volunteers undergoing CHMI; CS, circumsporozoite protein; ME-TRAP, multiple epitope–thrombospondin-related adhesion protein; MVA, modified vaccinia virus Ankara; SP, sterile protection.

Exploratory analysis of parasite densities by using area under the curve (AUC) analysis showed that parasite density over the ﬁrst 3 replication cycles in infected volunteers was a signiﬁcant predictor of the time to treatment (HR, 1.000015 [95% CI, 1.000008–1.000022], by Cox proportional hazards regression analysis; *P* < .000; Figure 6). Over the first, second, and third blood-stage replication cycles, there was a signiﬁcant reduction in parasite densities among ChAd63-MVA ME-TRAP vaccinees, as measured by AUC analysis (ie, log [parasite density + 1]), compared with unvaccinated controls, when vaccinees who achieved sterile protection were included in the analysis (cycle 1, *P* = .01; cycle 2, *P* = .03; and cycle 3 *P* = .05; by the 2-tailed *t* test, for all comparisons). Parasite densities in vaccinees receiving ChAd63 CS were significantly less than those in controls over the first blood-stage replication cycle only (*P* = .05 log [parasite density + 1], by the 2-tailed *t* test). AUC analysis showed that, compared with controls, ChAd63-MVA ME-TRAP resulted in a 79% reduction in parasitemia during cycle 1, whereas ChAd63-MVA CS caused a 69% reduction.

### ChAd63-MVA Safety Among All Regimens Following Sporozoite Challenge

No unexpected clinical or laboratory AEs were observed in vaccinees after CHMI, and there was no significant difference in the number of AEs related to CHMI between groups (*P* = .72; Supplementary Figure 3*A*). The total duration of symptoms in volunteers with symptomatic malaria ranged from 1 to 19 days (median, 6 days), with no significant difference between groups (*P* = .33; Supplementary Figure 3*B*). There was no difference between groups in the time that individuals were symptomatic before treatment (*P* = .43; Supplementary Figure 3*C*) or the number of symptoms present at time of treatment (*P* = .65) in volunteers with a diagnosis of malaria (Supplementary Figure 3*D*). Two of the 33 volunteers (6%) in whom malaria was diagnosed after CHMI had no symptoms of malaria at diagnosis. Of the volunteers with a malaria diagnosis, 28 (85%) experienced at least 1 AE after challenge that was severe in intensity (Supplementary Figure 3*E*). One volunteer in group 1 was admitted for inpatient management of vomiting secondary to antimalarial therapy (atovaquone/proguanil) 1 day after malaria diagnosis and was discharged the next day with no sequelae. Blood samples obtained 9, 35, and 90 days after CHMI and within 24 hours of diagnosis demonstrated transient hematological and biochemical abnormalities at frequencies and severities expected following *P. falciparum* infection (Supplementary Figure 3*F*) [40].

### Associations Between Immunological Outcomes and Vaccine Efficacy

**Figure 7.**
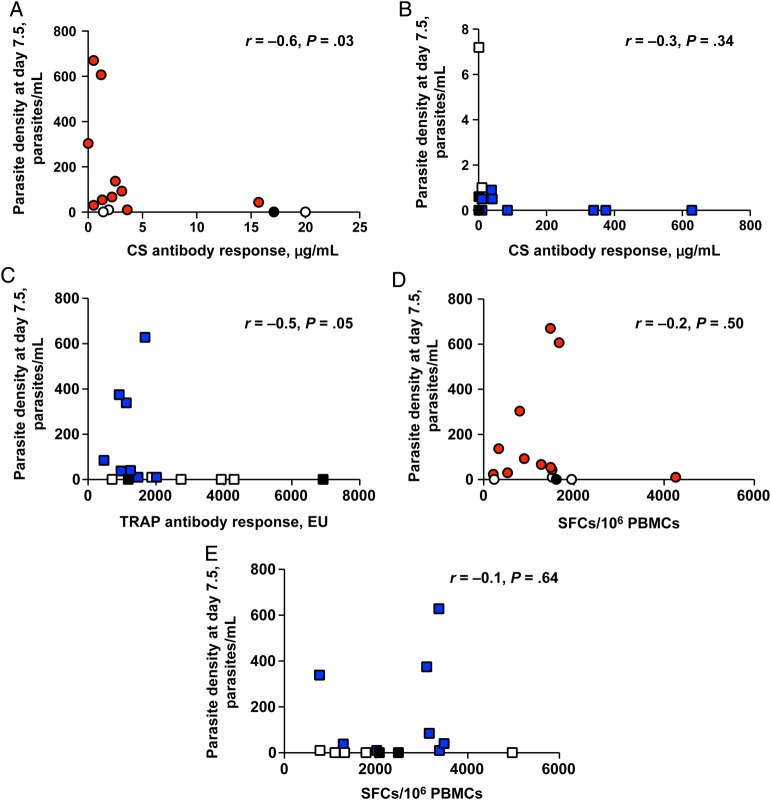
Associations between immunological outcomes and vaccine efficacy. Correlation between parasite density at day 7.5, measured by quantitative polymerase chain reaction (qPCR), and levels of anti–circumsporozoite protein (CS) immunoglobulin G (IgG) antibody in group 1 (CS; Spearman *r* = −0.6; *P* = .03; *A*) and group 2 (ME-TRAP; Spearman *r* = −.3*; P* = .34; *B*). *C*, Correlation between parasite density at day 7.5, measured by qPCR, and anti-TRAP IgG antibody responses in group 2 (ME-TRAP; Spearman *r* = −0.5; *P* = .05). *D*, Correlation between interferon γ (IFN-γ)–secreting T-cell frequency to CS measured by enzyme-linked immunosorbent spot (ELISPOT) parasite density at day 7.5 (parasite/mL measured by qPCR) in group 1 (CS; Spearman *r* = −0.2; *P* = .50. *E*, Correlation between IFN-γ–secreting T-cell frequency to ME-TRAP measured by ELISPOT and parasite density at day 7.5 (parasite/mL measured by qPCR) in group 2 (ME-TRAP; Spearman *r* = 0.1; *P* = .6). Abbreviations: Black filled points, sterilely protected vaccinees; EU, enzyme-linked immunosorbent assay units; group 1, ChAd63-MVA CS; group 2, ChAd63-MVA ME-TRAP; ME-TRAP, multiple epitope–thrombospondin-related adhesion protein; PBMC, peripheral blood mononuclear cell; SFC, spot-forming cell; unfilled points, vaccinees demonstrating delay to start of antimalarial therapy in comparison to unvaccinated control volunteers.

In group 1 but not group 2, IgG antibody responses to CS correlated significantly and negatively with qPCR-determined densities 7.5 days after CHMI (group 1: Spearman *r* = −0.6 [*P* = .03]; group 2: Spearman *r* = −0.3 [*P* = .34]; Figure 7*A* and 7*B*). A marginal negative correlation was seen in group 2 between IgG antibody responses to ME-TRAP and qPCR findings 7.5 days after CHMI (Spearman *r* = −0.5; *P* = .05; Figure 7*C*). No significant correlation was seen between IFN-γ ELISPOT findings for CS or ME-TRAP and qPCR findings 7.5 days after CHMI for group 1 or 2 (Figure 7*D* and 7*E*), in concordance with previous data in which ELISPOT-determined responses did not correlate with vaccine efficacy [17]. Phenotyping of the T-cell responses by flow cytometry was performed, and results will be reported in a subsequent article.

## DISCUSSION

In this first head-to-head comparison of the 2 leading preerythrocytic antigens, ME-TRAP and CS, delivered in the same vaccine platform, ME-TRAP had greater clinical efficacy, with sterile protection achieved in 13% of vaccinees (2 of 15) and a delayed time to diagnosis in 33% (5 of 15). This efficacy is slightly less than that recently reported in another CHMI study of ChAd63-MVA ME-TRAP [17], despite the induction of similar, very high frequency of antigen-specific T cells (peak median IFN-γ–secreting T cell count, 2027 in this study vs 2436 SFCs/million PBMCs in the previous study). Because the median time to diagnosis for unvaccinated control volunteers in this study was 1.5 days shorter than that of the previously reported CHMI study [17], it is possible that a larger challenge inoculum in this CHMI study could explain the small, suggested difference in efficacy results (there were no other differences in study methods). By the same reasoning, this could mean that the efficacy attained with ChAd63-MVA CS (sterile protection was achieved in 7% [1 of 15], and a delayed time to diagnosis was achieved by 20% [3 of 15]) underestimates that which may have been seen under less stringent CHMI conditions. Indeed, given that the infectious dose experienced by individuals in malaria-endemic countries is generally considerably less than that administered in CHMI studies [23], efficacy may prove to be greater in field studies.

ChAd63-MVA CS induced moderate to high IFN-γ–expressing T-cell responses, but anti-CS IgG levels were markedly lower than that seen with in a sporozoite CHMI trial assessing RTS,S, in which 50% of vaccinees (18 of 36) receiving RTS,S/AS01B and 32% (14 of 44) receiving RTS,S/AS02A achieved sterile protection (2.1 µg/mL with ChAd63 MVA CS vs 144 mg/mL with RTS,S/AS01B and 83 mg/mL with RTS,S/AS02A) [41]. The correlation between anti-CS antibodies and time to treatment suggests this may, surprisingly, be contributing to the mechanism of efficacy even at very low levels. This study provides the first evidence that sterile immunity can be generated with viral vectors encoding CS alone [41], although it is notable that some sterile efficacy has been reported using combinations of DNA and adenoviral vectors encoding CS and AMA1 [18].

Kaplan–Meier analysis of time to diagnosis between vaccinees and unvaccinated controls and numerous analyses of the qPCR data demonstrated significant efficacy for ChAd63-MVA ME-TRAP alone. There was no such statistically significant difference for the ChAd63-MVA CS vaccines using the same analysis. However, the AUC analysis, comparison of parasitemia at 7.5 days after CHMI, the evidence of sterile protection, and a delay to diagnosis in certain vaccinees all support the view that ChAd63-MVA CS led to a reduction (by approximately 69%–79%, depending on the analysis) in the number of parasites released from the liver. Because ChAd63-MVA ME-TRAP was, by use of the same measures, estimated to reduce the liver parasite burden by 79%–84%, it appears that relatively large reductions in liver-stage infection are required to significantly influence clinical outcomes after mosquito bite CHMI, as suggested previously [34, 35]. As this study shows, it can be difficult to quantify the efficacy of preerythrocytic vaccines that do not provide sterile immunity. We would argue that, given the necessarily small numbers of participants in CHMI studies and the importance of CHMI studies to deselect novel vaccine strategies and antigens [23], detailed analysis of qPCR data should be routinely performed to ensure that promising signals suggestive of clinically important efficacy are correctly identified.

Our data, importantly, compare the efficacy of ChAd63-MVA containing CS or ME-TRAP and, together with previous data comparing these antigens in DNA-MVA [25] and fowlpox-MVA regimes [26, 27, 42], support ME-TRAP as currently the most promising liver-stage antigen for inclusion in a future multistage vaccine. However, given the efficacy we have demonstrated here and the possibility that immunization with ME-TRAP and CS could prove to be more efficacious than either antigen alone, our next priority is to clinically assess the combination of ChAd63-MVA ME-TRAP and ChAd63-MVA CS in a CHMI trial.

We suggest that detailed analyses of parasite kinetics should be routinely performed in future CHMI vaccine studies to allow detection of smaller but biologically important differences in vaccine efficacy that could influence future vaccine development.

## Supplementary Data

Supplementary materials are available at *The Journal of Infectious Diseases* online (http://jid.oxfordjournals.org). Supplementary materials consist of data provided by the author that are published to benefit the reader. The posted materials are not copyedited. The contents of all supplementary data are the sole responsibility of the authors. Questions or messages regarding errors should be addressed to the author.

Supplementary Data
