# Supplementary material for: Evaluation of the Efficacy of ChAd63-MVA Vectored Vaccines Expressing Circumsporozoite Protein and ME-TRAP Against Controlled Human Malaria Infection in Malaria-Naive Individuals
Source: J Infect Dis. 2014 Oct 21;211(7):1076–86. doi: 10.1093/infdis/jiu579 (PMC4354983; doi:10.1093/infdis/jiu579)
Supplement: Supplementary Data [file supp_jiu579_jiu579supp_figs.pdf]

Figure S1: Adverse Events deemed possibly, probably or definitely related to administration of ChAd63 or MVA vectored vaccines (Groups 1 and 2). Only the highest intensity of each AE per subject is listed. There were no immunization-related serious adverse events. **(A)** Local AEs after administration of ChAd63 CS (Group 1) and ChAd63 ME-TRAP (Group 2). The "Other" local AE in Group 1 was mild vaccine site paraesthesia. **(B)** Local AEs after administration of MVA CS (Group 1) and MVA ME-TRAP (Group 2). The "Other" local AEs in Group 1 was mild vaccine site bruising. **(C)** Systemic AEs after administration of ChAd63 CS (Group 1) and ChAd63 ME-TRAP (Group 2). "Other" systemic AEs in Group 1 were mild insomnia, diarrhoea, abdominal cramps, neck pain, upper back pain, leucopenia (3.3 x 10^9/l), elevated ALT (70 IU/l), and moderate abdominal pain and lymphopenia (0.91 x 10^9/l). "Other" systemic AEs in Group 2 were mild rash, pharyngitis and coryzal symptoms. **(D)** Systemic AEs after administration of MVA CS (Group 1) and MVA ME-TRAP (Group 2). "Other" systemic AEs in Group 1 were mild light-headedness, disorientation, bruising at injection site, leucopenia (3.18 x 10^9/l) and episode of vasovagal syncope. The "Other" systemic AE in Group 2 was a mild rash. AE = adverse event; CS = circumsporozoite protein; ChAd63 = chimpanzee adenovirus 63; ME-TRAP = Multiple epitope-thrombospondin related adhesion protein; MVA = Modified Vaccinia virus Ankara.

Figure S2: Hierarchy of responses to peptide pools within each antigen and TRAP epitope mapping by IFNγ ELISPOT. **(A)** Responses to peptide pools in Group 1 (ChAd63-MVA CS) to CS (n=15). **(B)** Response to peptide pools in Group 2 (ChAd63-MVA ME-TRAP) to TRAP (n=15). **(C)** and **(D)** Responses to individual TRAP peptides in HLA-A3 positive volunteers in Group 2 (n=15) seven days after CHMI were mapped. Frequently recognized peptides are highlighted in boxes. **(E)** Responses to individual ME peptides in HLA-A3 positive volunteers in Group 2 (n=15) seven days after CHMI were mapped. **(F)** ELISPOT responses to 3 TRAP peptides stratified according to expression of the MHC class I allele A3 seven days after CHMI (n=11 per group). Data are from group 2 and an additional cohort of vaccinees from an identical previous vaccine trial using ChAd63 and MVA ME-TRAP[17] (*two-way ANOVA with Bonferroni post-test, 51-70 * = p<0.05, 141-160 and 151-170 *** = p<0.0001*). CS = Circumsporozoite protein;

ME-TRAP = Multiple-epitope thrombospondin-related adhesion protein; SFC = spot forming colonies per million peripheral blood mononuclear cells (PBMC); D28 = 4 weeks post simian adenovirus 63 (ChAd63) vaccination; D63 = 7 days post modified vaccinia virus Ankara (MVA) vaccination; C-1 = day before challenge.

Figure S3: Analysis of Controlled Human Malaria Infection Clinical Data. (A) Comparison of the number of AEs deemed possibly, probably or definitely related to malaria infection in individuals diagnosed with malaria in Group 1 (CS; *n=14;* mean 10.0, median 11.5) and Group 2 (ME-TRAP; *n=13*; mean 10.3, median 10.0) and controls (*n=6;* mean 9.5, median 9.5). *p=0.720; Kruskal-Wallis test.* **(B)** Comparison of the duration of symptoms (in days) deemed possibly, probably or definitely related to malaria infection in individuals diagnosed with malaria in Group 1 (CS; *n=14;* mean 6.4, median 6.0) and Group 2 (ME-TRAP; *n=13;* mean 7.4, median 6.0) and controls (*n=6* mean 8.7, median 9.0). *p=0.333*; Kruskal-Wallis test. **(C)** Comparison of the number of symptomatic days before malaria diagnosis between vaccinees in Group 1 (CS; *n=14*; mean 2.2, median 2.0) and Group 2 (ME-TRAP; n=13; mean 1.7, median 1.0) who underwent CHMI and were diagnosed with malaria and controls (*n=6;* mean 2.2, median 2.0). *p=0.428;* Kruskal-Wallis test: **(D)** Comparison of the number of symptoms at malaria diagnosis between vaccinees in Group 1 (CS; *n=14*; mean 6.8, median 7.0) and Group 2 (ME-TRAP; *n=13*; mean 8.0, median 9.0) and controls (*n=6;* mean 7.0, median 8.0). *p=0.654; Kruskal-Wallis test.* **(E)** Comparison of maximum severity of any symptom of malaria infection between vaccinees diagnosed with malaria in Group 1 (CS *n=14*), Group 2 (ME-TRAP *n=13*) and controls (*n=6*). **(F)** Laboratory AEs after CHMI deemed possibly, probably or definitely related to *P. falciparum* infection. For "any laboratory abnormality" only the highest intensity AE per subject is counted. On all groups median value is represented by a straight line through each plot; Group 1 = ChAd63-MVA CS; Group 2 = ChAd63 ME-TRAP; Controls = unvaccinated volunteers undergoing CHMI. AE = adverse event; CHMI = controlled human malaria infection. CS = circumsporozoite protein; ME-TRAP = Multiple epitope-thrombospondin related adhesion protein.

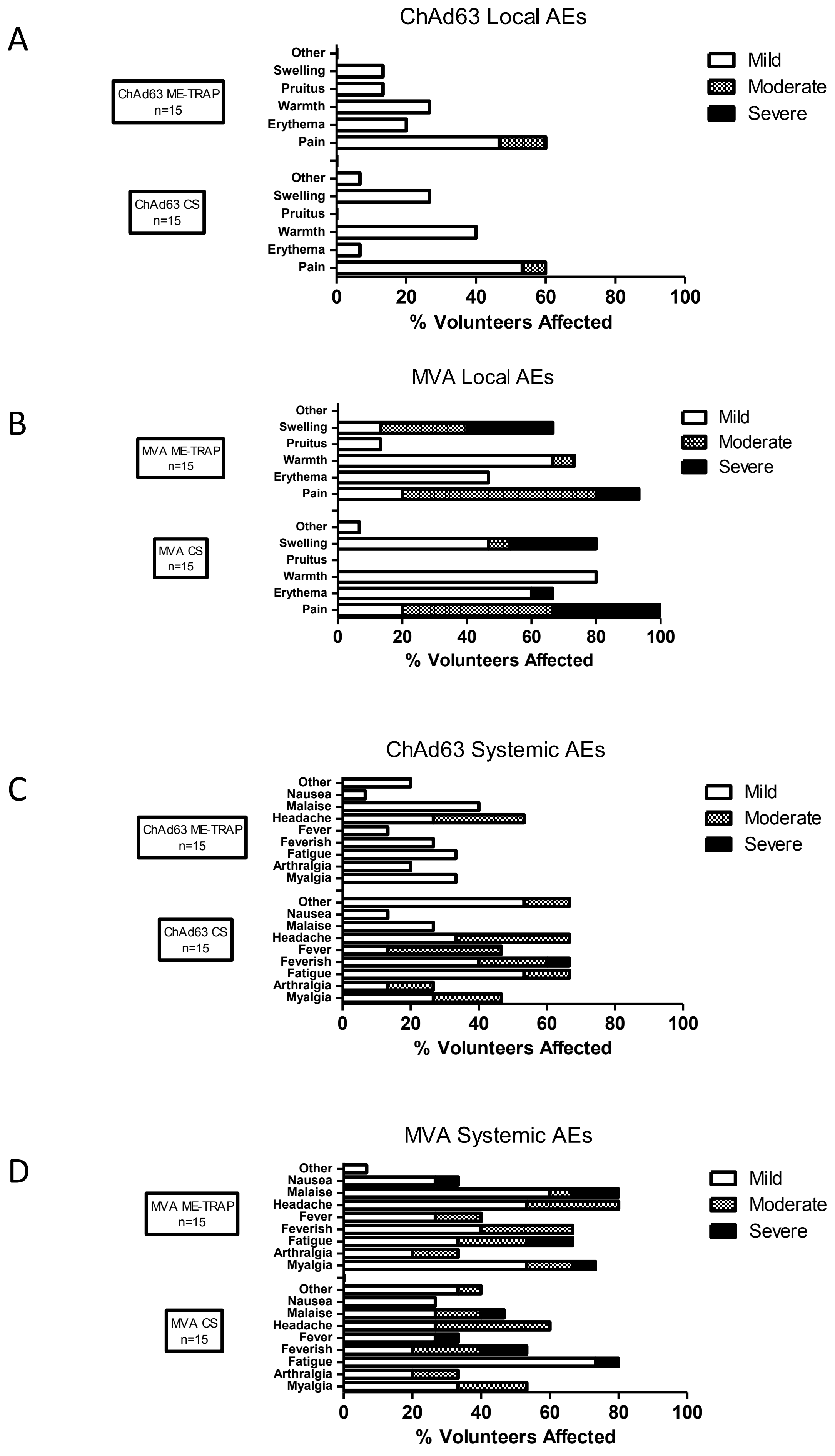
A
ChAd63 Local AEs
ChAd63 ME-TRAP
n=15
ChAd63 CS
n=15
Other
Swelling
Pruitus
Warmth
Erythema
Pain
Mild
Moderate
Severe
0
20
40
60
80
100
% Volunteers Affected
B
MVA Local AEs
MVA ME-TRAP
n=15
MVA CS
n=15
Other
Swelling
Pruitus
Warmth
Erythema
Pain
Mild
Moderate
Severe
0
20
40
60
80
100
% Volunteers Affected
C
ChAd63 Systemic AEs
ChAd63 ME-TRAP
n=15
ChAd63 CS
n=15
Other
Nausea
Malaise
Headache
Fever
Feverish
Fatigue
Arthralgia
Myalgia
Mild
Moderate
Severe
0
20
40
60
80
100
% Volunteers Affected
D
MVA Systemic AEs
MVA ME-TRAP
n=15
MVA CS
n=15
Other
Nausea
Malaise
Headache
Fever
Feverish
Fatigue
Arthralgia
Myalgia
Mild
Moderate
Severe
0
20
40
60
80
100
% Volunteers Affected

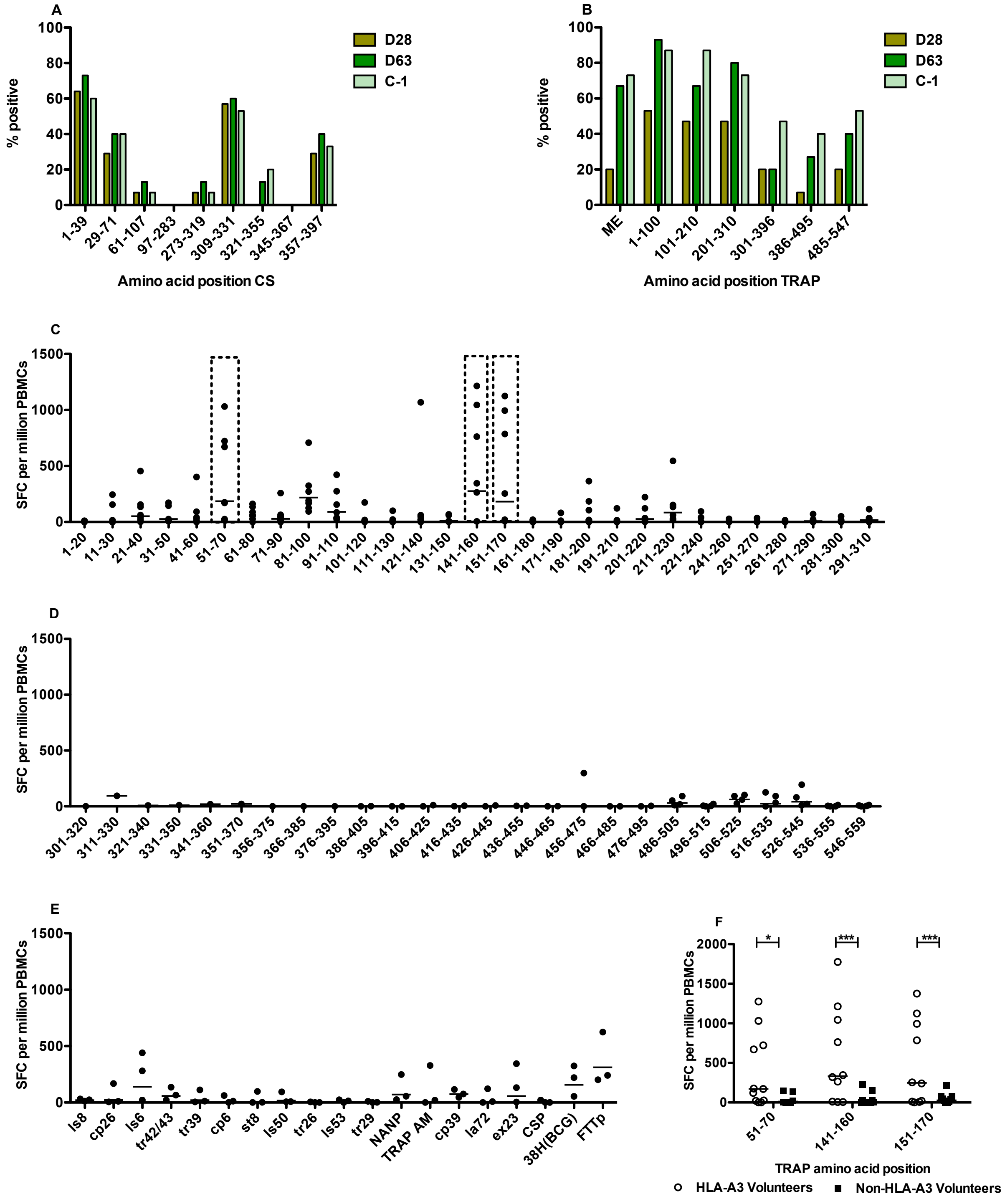
A
% positive
D28
D63
C-1
1-39
29-71
61-107
97-283
273-319
309-331
321-355
345-367
357-397
Amino acid position CS
B
% positive
D28
D63
C-1
ME
1-100
101-210
201-310
301-396
386-495
485-547
Amino acid position TRAP
C
SFC per million PBMCs
1-20
11-30
21-40
31-50
41-60
51-70
61-80
71-90
81-100
91-110
101-120
111-130
121-140
131-150
141-160
151-170
161-180
171-190
181-200
191-210
201-220
211-230
221-240
241-260
251-270
261-280
271-290
281-300
291-310
D
SFC per million PBMCs
301-320
311-330
321-340
331-350
341-360
351-370
356-375
366-385
376-395
386-405
396-415
406-425
416-435
426-445
436-455
446-465
456-475
466-485
476-495
486-505
496-515
506-525
516-535
526-545
536-555
546-559
E
SFC per million PBMCs
ls8
cp26
ls6
tr42/43
tr39
cp6
st8
ls50
tr26
ls53
tr29
NANP
TRAP AM
cp39
la72
ex23
CSP
38H(BCG)
FTTp
F
SFC per million PBMCs
*
***
***
51-70
141-160
151-170
TRAP amino acid position
HLA-A3 Volunteers
Non-HLA-A3 Volunteers

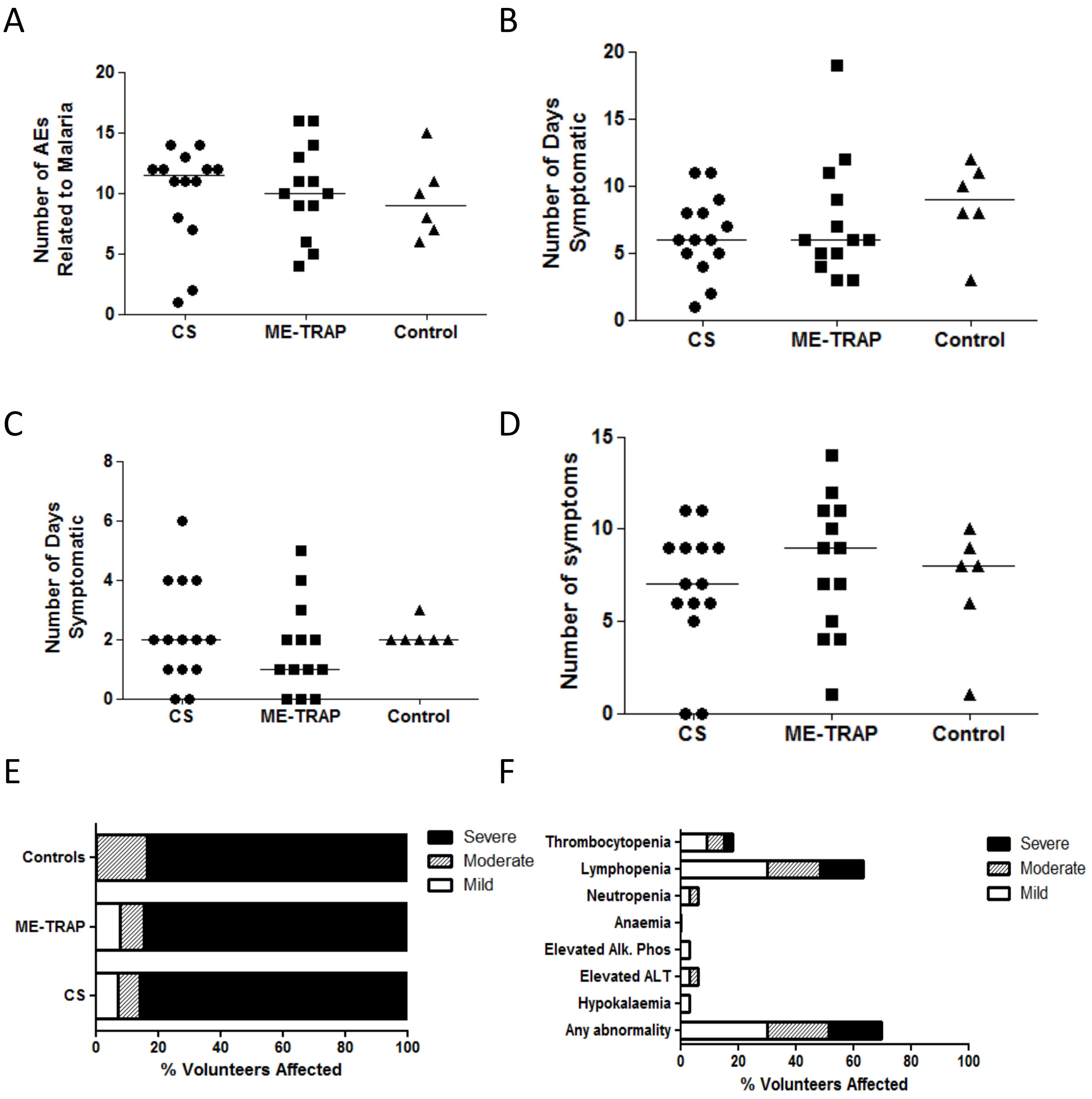
A
Number of AEs Related to Malaria
20
15
10
5
0
CS
ME-TRAP
Control
B
Number of Days Symptomatic
20
15
10
5
0
CS
ME-TRAP
Control
C
Number of Days Symptomatic
8
6
4
2
0
CS
ME-TRAP
Control
D
Number of symptoms
15
10
5
0
CS
ME-TRAP
Control
E
Controls
ME-TRAP
CS
0
20
40
60
80
100
% Volunteers Affected
Severe
Moderate
Mild
F
Thrombocytopenia
Lymphopenia
Neutropenia
Anaemia
Elevated Alk. Phos
Elevated ALT
Hypokalaemia
Any abnormality
0
20
40
60
80
100
% Volunteers Affected
Severe
Moderate
Mild
