# Supplementary material for: Evaluation of the Efficacy of ChAd63-MVA Vectored Vaccines Expressing Circumsporozoite Protein and ME-TRAP Against Controlled Human Malaria Infection in Malaria-Naive Individuals
Source: J Infect Dis. 2014 Oct 21;211(7):1076–86. doi: 10.1093/infdis/jiu579 (PMC4354983; doi:10.1093/infdis/jiu579)
Supplement: Supplementary Data [file supp_jiu579_jiu579supp.docx]

**SUPPLEMENTARY INFORMATION**

**Inclusion Criteria**

- Healthy adults aged 18 to 45 years.
- Able and willing (in the Investigator’s opinion) to comply with all study requirements.
- Willing to allow the investigators to discuss the volunteer’s medical history with their General Practitioner.
- Women only: Practice continuous effective contraception for the duration of the study.
- Agreement to refrain from blood donation during the course of the study and for at least 3 years after the end of their involvement in the study.
- Written informed consent to participate in the trial.
- Reachable (24/7) by mobile telephone during the period between CHMI and completion of antimalarial treatment.
- Willingness to take a curative anti-malaria regimen following CHMI.
- For volunteers not living in Oxford: agreement to stay in a hotel room close to the trial centre during a part of the study (from at least day 6.5 post mosquito bite until anti-malarial treatment is completed).
- Answer all questions on the informed consent quiz correctly.

### Exclusion Criteria

- History of clinical malaria (any species).
- Travel to a malaria endemic region during the study period or within the preceding six months with significant risk of malaria exposure.
- Use of systemic antibiotics with known antimalarial activity within 30 days of CHMI (e.g. trimethoprim-sulfamethoxazole, doxycycline, tetracycline, clindamycin, erythromycin, fluoroquinolones and azithromycin)
- Receipt of an investigational product in the 30 days preceding enrolment, or planned receipt during the study period.
- Prior receipt of an investigational malaria vaccine or any other investigational vaccine likely to impact on interpretation of the trial data.
- Any confirmed or suspected immunosuppressive or immunodeficient state, including HIV infection; asplenia; recurrent, severe infections and chronic (more than 14 days) immunosuppressant medication within the past 6 months (inhaled and topical steroids are allowed).
- Use of immunoglobulins or blood products within 3 months prior to enrolment.
- History of allergic disease or reactions likely to be exacerbated by any component of the vaccine (e.g. egg products, Kathon) or malaria infection.
- Any history of anaphylaxis post vaccination.
- History of clinically significant contact dermatitis.
- History of sickle cell anaemia, sickle cell trait, thalassemia or thalassemia trait or any haematological condition that could affect susceptibility to malaria infection.
- Pregnancy, lactation or intention to become pregnant during the study.
- Contraindications to the use of all three proposed anti-malarial medications; Malarone^®^, Riamet^®^ and Chloroquine.
- History of cancer (except basal cell carcinoma of the skin and cervical carcinoma in situ).
- History of serious psychiatric condition that may affect participation in the study.
- Any other serious chronic illness requiring hospital specialist supervision.
- Suspected or known current alcohol abuse as defined by an alcohol intake of greater than 42 units every week.
- Suspected or known injecting drug abuse in the 5 years preceding enrolment.
- Seropositive for hepatitis B surface antigen (HBsAg).
- Seropositive for hepatitis C virus (antibodies to HCV) with positive PCR for hepatitis C at screening.
- An estimated, ten-year risk of fatal cardiovascular disease of ≥5%, as estimated by the Systematic Coronary Risk Evaluation (SCORE) system. [[1](#_ENREF_1)]
- Positive family history in 1st and 2nd degree relatives < 50 years old for cardiac disease.
- Volunteers unable to be closely followed for social, geographic or psychological reasons.
- Any clinically significant abnormal finding on biochemistry or haematology blood tests, urinalysis or clinical examination.
- Any other significant disease, disorder or finding which may significantly increase the risk to the volunteer because of participation in the study, affect the ability of the volunteer to participate in the study or impair interpretation of the study data.

### Re-vaccination exclusion criteria

The following AEs associated with any vaccine constituted absolute contraindications to further administration of an IMP to the volunteer in question.

- Anaphylactic reaction following administration of vaccine
- Pregnancy

The following adverse events constituted contraindications to administration of vaccine at that point in time; if any one of these adverse events occurs at the time scheduled for vaccination, the subject may be vaccinated at a later date, or withdrawn, at the discretion of the investigator;

- Acute disease at the time of vaccination. (Acute disease is defined as the presence of a moderate or severe illness with or without fever.) All vaccines can be administered to persons with a minor illness such as diarrhoea, mild upper respiratory infection with or without low-grade febrile illness, *i.e.*, temperature of <37.5°C (99.5°F).
- Temperature of ≥37.5°C (99.5°F) at the time of vaccination.

Exclusion Criteria on Day of Controlled Human Malaria Infection (CHMI)

The following constituted absolute contraindications to CHMI;

- Acute disease, defined as moderate or severe illness with or without fever.
- Pregnancy.

**Vaccinations**

15 volunteers in Group 1 were vaccinated with ChAd63 CS 5 x 10^10^ viral particles (vp) (undiluted and administered in 354µL) followed 8 weeks later by MVA CS 2 x 10^8^ plaque forming units (pfu) (undiluted and administered in 339µL). 15 volunteers in Group 2 were vaccinated with ChAd63 ME-TRAP 5 x 10^10^ viral particles (vp) (undiluted and administered in 347µL) followed 8 weeks later by MVA ME-TRAP 2 x 10^8^ plaque forming units (pfu) (undiluted and administered in 274µL).

**Clinical Follow-up & Safety Assessment**

All volunteers were observed in clinic for 30 minutes following each vaccination. Volunteers were given a digital thermometer, injection site reaction measurement tool and symptom diary card to record their daily temperature, injection site reactions and solicited systemic AEs for 7 days following each vaccination. Vaccinees attended for clinical review at days 1, 14, 28, 56, 57, 63 post ChAd63 vectored vaccines, days 57 and 63 post MVA vectored vaccines and the day before CHMI (C-1). A time window ranging between 12 hours and 7 days was allowed for vaccination and post vaccination follow-up visits. Local and systemic reactogenicity was evaluated at subsequent clinic visits and graded for severity, outcome and association to vaccination as previously described. [[2](#_ENREF_2)] Blood was sampled at all visits post vaccination. Post CHMI volunteers were reviewed on day 6 post CHMI in the evening (C+6.5) and then twice a day, morning and evening between C+7 and C+14. Undiagnosed volunteers were reviewed once a day in the morning between C+15 and C+21. At each visit, blood was sampled for microscopy & qPCR, physical observations performed and AEs solicited. On diagnosis, volunteers were treated with a 3-day curative course of oral Malarone^®^ where each dose was directly observed in clinic. Volunteers intolerant of Malarone^®^ were prescribed an appropriate alternative (oral Riamet^®^ or Cholorquine). Volunteers were reviewed 24 and 48 hours post diagnosis where blood was sampled for microscopy. Provided these two blood-films were negative for parasites, volunteers were not reviewed again in clinic until C+35. If one of these blood films were positive, volunteers continued to be reviewed in clinic at 24-hour intervals until two consecutive blood films were negative. Volunteers were then reviewed at C+35 and C+90 where safety assessments were conducted. Full blood count with differential, platelet count and serum biochemistry (including electrolytes, urea, creatinine, bilirubin, alanine aminotransferase, alkaline phosphatase and albumin) were measured at all visits before CHMI (except days 1 and 57), at visit C+9, within 24 hours of diagnosis, and at visits on C+35 and C+90. Blood was sampled for exploratory immunology studies at all visits before CHMI, C-1, C+7, C+14, C+21 (if persistently slide negative), within 48 hours of diagnosis, C+35 and C+90.

**Quantitative Real-Time PCR (qPCR)**

qPCR was conducted as previously described. [[3](#_ENREF_3)] Briefly, blood was filtered to reduce white cell content and DNA was extracted from filtered 0.5mL blood using Qiagen Blood Mini Kit. 10% of each extraction (total eluate volume = 50μL, with 5μL used per assay) was run in triplicate for qPCR – equivalent to 150μL blood directly assessed. Parasites per mL (p/mL) equivalent mean values were generated by a standard Taqman absolute quantitation, against a defined plasmid standard curve with an ABI StepOne Plus machine and v2.1 software. Default Universal qPCR and QC settings were used apart from the use of 45 cycles and 25μL reaction volume. Based upon results obtained using dilution series of microscopically-counted cultured parasites, this method has a lower limit of quantification (LLQ, defined as %CV<20%) of around 20 parasites/mL blood (p/mL).[[4](#_ENREF_4)] Counted parasite dilution series results suggest that the lower limit of probable detection (LLD, i.e. a probability of >50% of ≥1 positive result among three replicate PCR reactions) is in the region of 5 p/mL, while samples at 1 p/mL are consistently negative (24/24 PCR reactions). Positive results in this assay (even at very low level) are thus essentially 100% specific for genuine parasitaemia, with positive results beneath the LLQ likely to signify parasitaemia in the range 2-20p/mL.

For quality control purposes, qPCR samples were re-tested if;

- Replicates included a mixture of positive and negative (in terms of amplification) results with one or more positive results > 100 parasites/mL.
- The %CV of any results were high outliers.

All ‘passed’ data following quality control QC steps above, including any 0 values, were used to generate the mean for each time-point. For modelling purposes, when a mean value of < 20p/mL was obtained for a time-point, the following rules were applied;

- If occurring before at least 2 subsequent positive values (>20p/mL), this time-point was allocated the value of 10p/mL.
- If occurring following any sample >20p/mL this time-point was allocated the value of 10p/mL.
- In all other cases a negative value (0p/mL) was allocated.

***Ex-vivo* interferon-γ (IFN-γ) Enzyme-Linked Immunosorbent Spot (ELIspot)**

*Ex vivo* (18 hour stimulation) ELISPOT assays were performed using Multiscreen IP ELISPOT plates (Millipore), human IFNγ SA-ALP antibody kits (Mabtech) and BCIP NBT-plus chromogenic substrate (Moss Inc). Cells were cultured in RPMI (Sigma) containing 1% sterile filtered Penicillin-Streptomycin (Sigma), 1% L-Glutamine (Sigma) and 10% heat-inactivated, sterile-filtered foetal calf serum, previously screened for low reactivity (Labtech International). Antigens were tested in duplicate with 250,000 PBMC added to each well of the *ex vivo* ELISPOT plate. Plates were counted using an AID automated ELISPOT counter (AID Diagnostika GmbH, algorithm C), using identical settings for all plates and counts were adjusted only to remove artefacts. Responses to the negative control were always < 80 SFC per million PBMC. Responses were considered positive if four times greater than the negative control for the corresponding sample.

**Peptides for T cell Assays (Tables S4-S6)**

TRAP peptides were 20 amino acids in length, overlapping by 10 amino acids (Neopeptide), assayed in 6 pools of 7-10 peptides at 10 μg/mL. CSP peptides were 15 amino acids in length, overlapping by 11 amino acids (a kind gift of the Malaria Department, US Naval Medical Research Center), assayed in 9 pools of 3-12 peptides at 10 μg/mL. Responses were averaged across duplicates, responses in unstimulated (negative control) wells were subtracted and then responses in individual pools were summed for each strain of the TRAP antigen or for CSP. ME responses were assayed in a single pool and peptide pool configurations are shown in Supplementary Table S1 - S3. Staphylococcal enterotoxin B (0.02 μg/mL) and phytohaemmagglutinin-L (10μg/mL) were used as a positive control. Epitope mapping was performed using individual 20mer peptides spanning the length of the T9/96 TRAP protein in single ELISPOT wells, each containing 100,000 PBMC.

**Total IgG Enzyme Linked Immunosorbent Assay (ELISA) to TRAP**

Recombinant TRAP antigen was produced by transient transfection of HEK293E cells, using a method similar to that previously reported (*Hodgson et al. In press*). [[5](#_ENREF_5)] A transgene comprising the human tissue plasminogen activator secretory signal peptide fused in frame with the 3D7-clone TRAP ectodomain (lacking the native signal peptide, transmembrane domain and a run of PNP repeats stretching from P356 to P370) was codon-optimised for mammalian expression (Life Technologies). The transgene cassette was cloned using the InFusion enzyme system (ClonTech) into the pENTR LPTOS plasmid backbone, [[6](#_ENREF_6)] in which expression of the transgene is driven by an intron-containing CMV immediate early promoter, with additional in-frame C-terminal biotin acceptor peptide and Strep(II) tags. [[7](#_ENREF_7)] Four days after polyethyleneimine mediated transfection of HEK293E cells, supernatant was harvested and affinity purified on a Streptactin affinity column (IBA Lifesciences). The resulting protein was >90% pure, as demonstrated by Coomassie Blue stained SDS-PAGE (data not shown).

Nunc-Immuno 96 well plates were coated with 0.5μg/mL of TRAP antigen in carbonate-bicarbonate coating buffer and left overnight at 4°C. Plates were washed 6x with PBS-Tween (PBS/T), then blocked with 1% BSA in PBS/T for 1 hour at room temperature (RT). Serum was diluted in PBS/T containing 0.2% BSA at concentrations of 1:100, 1:300, or 1:900, and added in triplicate. Serum samples from days 0, 28, 56, 63, C-1, C+7 and C+90 were analysed. Plates were incubated at RT for 2 hours then washed as before. A secondary antibody (goat anti-human whole IgG conjugated to alkaline phosphatase, Sigma) was added at a dilution of 1:1000 in PBS/T 0.2% BSA for 1 hour at RT. After a final wash, plates were developed by adding 4-nitrophenyl phosphate in diethanolamine buffer (Pierce).

A positive reference standard (made from pooled TRAP-positive serum) was used on each plate to give a standard curve. It was added in duplicate at an initial dilution of 1:100 (in PBS/T 0.2% BSA) and diluted 2-fold 10 times, starting with an arbitrary value of 20 antibody units. 4 blank wells (zero antibody units) were also designated. The optical density (OD) values were then fitted to a 4 parameter standard curve using SOFTmax PRO software^.^. [[8](#_ENREF_8)] An internal control was included on every plate in triplicate made up from a 1:400 dilution (in PBS/T 0.2% BSA) of the positive standard. OD was read at 405nm using an ELx800 microplate reader. Test sera antibody units were calculated from their OD values using the parameters estimated from the standard curve.

**Total IgG Enzyme Linked Immunosorbent Assay (ELISA) to CS** [[9](#_ENREF_9)]

ELISA 96-well plates were coated with a synthetic peptide (Eurogentec) based on the repeat region of the PfCSP with the amino acid sequence CS(NANP)6C. The peptide was coated at a concentration of 0.2 μg/mL in a volume of 100 μL per well. Plates were placed inside a humidity chamber and incubated overnight (16 - 20 h) at 22°C. Plates were washed four times with 1xPBS (pH 7.4) containing 0.5% Tween-20 and blocked with 0.5% casein blocking buffer (Sigma) for 1 h at 22°C. Plates were washed four times and serially diluted samples were added and incubated at 22°C for 2 h. After washing four times, peroxidase labeled goat anti-human IgG (KPL) was added at a dilution of 1:4,000 and incubated at 22°C for 1 h. After washing four times, ABTS Peroxidase substrate (KPL) was added for development and incubated for 1 h at 22°C. The data were collected using Softmax Pro GXP, data were fit to a 4-parameter logistic curve, and the serum dilution at which the optical density was 1.0 (OD 1.0) calculated. To serve as a positive control, serum obtained from a volunteer participating in a Phase 1/2a challenge trial of R32NS181 formulated with alum was used. [[10](#_ENREF_10)] The individual had anti-PfCSP antibodies but was not protected (*personal communication from WRAIR serology laboratory*). Samples were considered positive if the difference between the post-immunization OD 1.0 and the pre-immunization OD 1.0 (net OD 1.0) was > 50 and the ratio of post- immunization OD 1.0 to pre-immunization OD 1.0 (ratio) was > 2. For example, if the OD 1.0 was 150 post-immunization and 50 pre-immunization, the net OD 1.0 would be 100, and the ratio of OD 1.0 post-immunization to OD 1.0 pre-immunization would be 3. This would be considered positive.

**Results of Mapping of dominant peptides within peptide pools (Figure S3)**

Detailed mapping of T cell responses to CS peptides was not performed as this has been described recently in great detail along with several HLA class I-restricted epitopes. [[11](#_ENREF_11)] Seven of the nine CS peptide pools were recognised after vaccination with ChAd63 and MVA representing 77% of the antigen (*Figure S3A*). Two of the peptide pools spanning the CSP antigen (Cp1 and Cp6) were recognised in at least 50% of volunteers 28 days after priming with ChAd63 CS (D28), 7 after boosting with MVA CS (D63) and the day before CHMI (C-1). All ME-TRAP peptide pools were recognised by at least one volunteer at each time point with the 3 pools spanning the first half of the antigen showing immunodominance (*Figure S3B*). The ME string was much less immunogenic than TRAP with responses > 200 spot forming cells per million peripheral blood mononuclear cells (*Figure S3C*) detected to only 4 peptides, all within the same vaccinee**.**

Responses to individual constituent 20mer peptides of each ME-TRAP peptide pool were assessed by *ex vivo* IFNy ELISpot seven days after CHMI in those volunteers where responses were > 200 SFC to the pool at the time of challenge. In a previous challenge study, [[12](#_ENREF_12)] all protected volunteers expressed an HLA-A3 MHC allele and so to investigate any possible protective effect of HLA-A3-restriected epitopes, mapping was restricted to HLA-A3 positive volunteers. A number of different epitopes were identified, with the majority of responses being to the first half of the TRAP sequence (*Figure S3B*). The most frequently recognised peptides (and those with the highest responses amongst these volunteers) corresponded to amino acids 51-70, 81-100, 141-160 and 151-170 (*Figure S3C and S3D*). Responses were of limited magnitude to epitopes in the ME pool (*Figure S3E*).

The ELISpot responses in HLA-A3 volunteers in Group 2 were also compared to non-HLA-A3 volunteers in a previous clinical trial who received the same vaccination regimen. [[12](#_ENREF_12)] Responses to the peptides corresponding to amino acids 51-70, 141-160 and 151-170 were significantly greater in HLA-A3 volunteers compared to non-HLA-A3 volunteers (*two-way ANOVA with Bonferroni post-test, 51-70 p<0.05, 141-160 and 151-170 p<0.0001, Figure S3F*). However, there was a high degree of promiscuity to most epitopes, with those identified in HLA-A3 volunteers also present in non-HLA-A3 volunteers. Furthermore, we observed that previously identified HLA-A2 restricted epitopes from TRAP, such as amino acids 121-140, [[13](#_ENREF_13)] were also responsive in non-HLA-A2 volunteers (for example, in a HLA-A3/A26 positive volunteer) after vaccination with the ChAd63-MVA regimen.

**SUPPLEMENTARY TABLES**

**Table S1: Criteria for Assessing Relationship of AE to study intervention**

| 0 | **No Relationship** | No temporal relationship to study product ***and***  Alternate aetiology (clinical state, environmental or other interventions); ***and***  Does not follow known pattern of response to study product |
| --- | --- | --- |
| 1 | **Unlikely** | Unlikely temporal relationship to study product ***and***  Alternate aetiology likely (clinical state, environmental or other interventions) ***and***  Does not follow known typical or plausible pattern of response to study product. |
| 2 | **Possible** | Reasonable temporal relationship to study product; ***or***  Event not readily produced by clinical state, environmental or other interventions; ***or***  Similar pattern of response to that seen with other vaccines |
| 3 | **Probable** | Reasonable temporal relationship to study product; ***and***  Event not readily produced by clinical state, environment, or other interventions ***or***  Known pattern of response seen with other vaccines |
| 4 | **Definite** | Reasonable temporal relationship to study product; ***and***  Event not readily produced by clinical state, environment, or other interventions; ***and***  Known pattern of response seen with other vaccines |

**Table S2: Severity grading criteria for injection site pain, erythema and swelling.**

| **Adverse Event** | **Grade** | **Intensity** |
| --- | --- | --- |
| Pain at injection site | 1 | Pain that is easily tolerated |
|  | 2 | Pain that interferes with daily activity |
|  | 3 | Pain that prevents daily activity |
| Erythema at injection site* | 1 | >3 - ≤50 mm |
|  | 2 | >50 - ≤100 mm |
|  | 3 | >100 mm |
| Swelling at injection site | 1 | >0 - ≤20 mm |
|  | 2 | >20 - ≤50 mm |
|  | 3 | >50 mm |

**erythema ≤3mm is an expected consequence of skin puncture and will therefore not be considered an adverse event*

**Table S3: Severity Grading Criteria for AEs**

| **GRADE 0** | None |
| --- | --- |
| **GRADE 1** | Mild: Transient or mild discomfort (< 48 hours); no medical intervention/therapy required |
| **GRADE 2** | Moderate: Mild to moderate limitation in activity - some assistance may be needed; no or minimal medical intervention/therapy required |
| **GRADE 3** | Severe: Marked limitation in activity, some assistance usually required; medical intervention/therapy required, hospitalisation possible |

**Table S4: TRAP peptide pool format**

| **Peptide Name** | **Peptide sequence T9/96** | **Peptide sequence 3D7** | **T9/96 Peptide Pool** | **3D7 Peptide Pool** |
| --- | --- | --- | --- | --- |
| TRAP-1 | MNHLGNVKYLVIVFLIFFDL |  | TT1-10 | TD1-10 |
| TRAP-2 | VIVFLIFFDLFLVNGRDVQN |  | TT1-10 | TD1-10 |
| TRAP-3 | FLVNGRDVQNNIVDEIKYSE | **FLVNGRDVQNNIVDEIKYRE** | TT1-10 | TD1-10 |
| TRAP-4 | NIVDEIKYSEEVCNDQVDLY | **NIVDEIKYREEVCNDEVDLY** | TT1-10 | TD1-10 |
| TRAP-5 | EVCNDQVDLYLLMCSGSIR | **EVCNDEVDLYLLMCSGSIR** | TT1-10 | TD1-10 |
| TRAP-6 | LLMCSGSIRRHNWVNHAVP |  | TT1-10 | TD1-10 |
| TRAP-7 | RHNWVNHAVPLAMKLIQQLN |  | TT1-10 | TD1-10 |
| TRAP-8 | LAMKLIQQLNLNDNAIHLYV | **LAMKLIQQLNLNDNAIHLYA** | TT1-10 | TD1-10 |
| TRAP-9 | LNDNAIHLYVNVFSNNAKEI | **LNDNAIHLYASVFSNNAREI** | TT1-10 | TD1-10 |
| TRAP-10 | LNDNAIHLYVNVFSNNAKEI | **SVFSNNAREIIRLHSDASKN** | TT1-10 | TD1-10 |
| TRAP-11 | IRLHSDASKNKEKALIIIRS | **IRLHSDASKNKEKALIIIKS** | TT11-20 | TD11-20 |
| TRAP-12 | KEKALIIIRSLLSTNLPYGR | **KEKALIIIKSLLSTNLPYGK** | TT11-20 | TD11-20 |
| TRAP-13 | LLSTNLPYGRTNLTDALLQV | **LLSTNLPYGKTNLTDALLQV** | TT11-20 | TD11-20 |
| TRAP-14 | TNLTDALLQVRKHLNDRINR |  | TT11-20 | TD11-20 |
| TRAP-15 | RKHLNDRINRENANQLVVIL |  | TT11-20 | TD11-20 |
| TRAP-16 | ENANQLVVILTDGIPDSIQD |  | TT11-20 | TD11-20 |
| TRAP-17 | TDGIPDSIQDSLKESRKLSD |  | TT11-20 | TD11-20 |
| TRAP-18 | SLKESRKLSDRGVKIAVFGI |  | TT11-20 | TD11-20 |
| TRAP-19 | RGVKIAVFGIGQGINVAFNR |  | TT11-20 | TD11-20 |
| TRAP-20 | GQGINVAFNRFLVGCHPSDG |  | TT11-20 | TD11-20 |
| TRAP-21 | FLVGCHPSDGKCNLYADSAW |  | TT21-30 | TD21-30 |
| TRAP-22 | KCNLYADSAWENVKNVIGPF |  | TT21-30 | TD21-30 |
| TRAP-23 | ENVKNVIGPFMKAVCVEVEK |  | TT21-30 | TD21-30 |
| TRAP-24 | MKAVCVEVEKTASCGVWDEW |  | TT21-30 | TD21-30 |
| TRAP-25 | TASCGVWDEWSPCSVTCGKG |  | TT21-30 | TD21-30 |
| TRAP-26 | SPCSVTCGKGTRSRKREILH |  | TT21-30 | TD21-30 |
| TRAP-27 | TRSRKREILHEGCTSEIQEQ | **TRSRKREILHEGCTSELQEQ** | TT21-30 | TD21-30 |
| TRAP-28 | EGCTSEIQEQCEEERCPPKW | **EGCTSELQEQCEEERCLPKR** | TT21-30 | TD21-30 |
| TRAP-29 | CEEERCPPKWEPLDVPDEPE | **CEEERCLPKREPLDVPDEPE** | TT21-30 | TD21-30 |
| TRAP-30 | EPLDVPDEPEDDQPRPRGDN |  | TT21-30 | TD21-30 |
| TRAP-31 | DDQPRPRGDNSSVQKPEENI | **DDQPRPRGDNFAVEKPNENI** | TT31-40 | TD31-40 |
| TRAP-32 | SSVQKPEENIIDNNPQEPSP | **FAVEKPNENIIDNNPQEPSP** | TT31-40 | TD31-40 |
| TRAP-33 | IDNNPQEPSPNPEEGKDENP | **IDNNPQEPSPNPEEGKGENP** | TT31-40 | TD31-40 |
| TRAP-34 | NPEEGKDENPNGFDLDENPE | **NPEEGKGENPNGFDLDENPE** | TT31-40 | TD31-40 |
| TRAP-35 | NGFDLDENPENPPNPDIPEQ | **NGFDLDENPENPPNPPNPPN** | TT31-40 | TD31-40 |
| TRAP-36 | NPPNPDIPEQKPNIPEDSEK | **NPPNPPNPPNPPNPPNPPNP** | TT31-40 | TD31-40 |
| TRAP-37 | *NONE* | **PPNPPNPPNPDIPEQKPNIP** | TT31-40 | TD31-40 |
| TRAP-38 | DIPEQKPNIPEDSEKEVPSD | **DIPEQKPNIPEDSEKEVPSD** | TT31-40 | TD31-40 |
| TRAP-39 | EDSEKEVPSDVPKNPEDDRE |  | TT31-40 | TD31-40 |
| TRAP-40 | VPKNPEDDREENFDIPKKPE |  | TT31-40 | TD31-40 |
| TRAP-41 | ENFDIPKKPENKHDNQNNLP |  | TT41-50 | TD41-50 |
| TRAP-42 | NKHDNQNNLPNDKSDRNIPY | **NKHDNQNNLPNDKSDRYIPY** | TT41-50 | TD41-50 |
| TRAP-43 | NDKSDRNIPYSPLPPKVLDN | **NDKSDRYIPYSPLAPKVLDN** | TT41-50 | TD41-50 |
| TRAP-44 | SPLPPKVLDNERKQSDPQSQ | **SPLAPKVLDNERKQSDPQSQ** | TT41-50 | TD41-50 |
| TRAP-45 | ERKQSDPQSQDNNGNRHVPN |  | TT41-50 | TD41-50 |
| TRAP-46 | DNNGNRHVPNSEDRETRPHG |  | TT41-50 | TD41-50 |
| TRAP-47 | SEDRETRPHGRNNENRSYNR |  | TT41-50 | TD41-50 |
| TRAP-48 | RNNENRSYNRKYNDTPKHPE |  | TT41-50 | TD41-50 |
| TRAP-49 | KYNDTPKHPEREEHEKPDNN |  | TT41-50 | TD41-50 |
| TRAP-50 | REEHEKPDNNKKKGESDNKY |  | TT41-50 | TD41-50 |
| TRAP-51 | KKKGESDNKYKIAGGIAGGL |  | TT51-57 | TT51-57 |
| TRAP-52 | KIAGGIAGGLALLACAGLAY |  | TT51-57 | TT51-57 |
| TRAP-53 | ALLACAGLAYKFVVPGAATP |  | TT51-57 | TT51-57 |
| TRAP-54 | KFVVPGAATPYAGEPAPFDE |  | TT51-57 | TT51-57 |
| TRAP-55 | YAGEPAPFDETLGEEDKDLD |  | TT51-57 | TT51-57 |
| TRAP-56 | TLGEEDKDLDEPEQFRLPEE |  | TT51-57 | TT51-57 |
| TRAP-57 | EPEQFRLPEENEWN |  | TT51-57 | TT51-57 |

*Peptide sequences and residue numbers based on those of the P. falciparum clone T9/96 (GenBank no.* *CAA31440.1).*

**Table S5: ME peptide pool format** [[14](#_ENREF_14)]

| **Peptide Name** | **Peptide sequence** | **Antigen** |
| --- | --- | --- |
| st8 | MINAYLDKL | STARP |
| ls50 | ISKYEDEI | LSA1 |
| pb9 | SYIPSAEKI | *P. berghei CSP* |
| ls8 | KPNDKSLY | LSA1 |
| cp26 | KPKDELDY | CSP |
| ls6 | KPIVQYDNF | LSA1 |
| tr42/43 | ASKNKEKALII | TRAP |
| tr39 | GIAGGLALL | TRAP |
| cp6 | MNPNDPNRNV | CSP |
| tr26 | HLGNVKYLV | TRAP |
| ls53 | KSLYDEHI | LSA1 |
| tr29 | LLMCSGSI | TRAP |
| csp | DPNANPNVDPNANPNV | CSP |
| 38H BCG | QVHFQPLPPAVVKL | BCG |
| FTTp | QFIKANSKFIGITE | TT |
| cp39 | YLNKIQNSL | CSP |
| la72 | MEKLKELEK | LSA3 |
| ex23 | ATSVLAGL | EXP1 |
| nanp | NANPNANPNANPNANP | CSP |
| trapAM | DEWSPCSVTCGKGTRSRKRE | TRAP |

**Table S6: CS peptide pool format**

| **Pool** | **CSP aa** | **No. peptides** |
| --- | --- | --- |
| **Cp1** | **1-39** | **7** |
| **Cp2** | **29-71** | **8** |
| **Cp3** | **61-107** | **9** |
| **Cp4** | **97-283** | **12** |
| **Cp5** | **273-319** | **9** |
| **Cp6** | **309-331** | **3** |
| **Cp7** | **321-335** | **6** |
| **Cp8** | **345-367** | **3** |
| **Cp9** | **357-397** | **8** |

*Peptide sequences and residue numbers were based on those of the P. falciparum clone 3D7 (GenBank no. X15363). Series of 15 amino acid peptide sequences overlapping by 11 amino acids.*

**Table S7: Criteria for Start of Anti-Malaria Treatment**

|  | **THICK FILM MICROSCOPY** | |
| --- | --- | --- |
| **MALARIAL SYMPTOMS** | **Positive** | **Negative** |
| **Symptomatic** | Treatment started | Treatment started if any available PCR result is > 500 parasites/mL |
| **Asymptomatic** | Treatment started if any available PCR result is > 500 parasites/mL  (Otherwise delay treatment) | Treatment not started |

**Table S8. Raw qPCR data (parasites/mL).** Top row represents day of follow-up visit post challenge. Data highlighted in red represent qPCR measurement on day of diagnosis for a particular volunteer. ND = Not detected. Highlighted in grey are samples positive but at < lower limit of detection (i.e. <20 parasites/ mL). VIN = Volunteer Identification Number. Group 1 = ChAd63-MVA CS vaccinees. Group 2 = ChAd63-MVA ME-TRAP vaccinees. Group 3 = Unvaccinated volunteers.

**SUPPLEMENTARY REFERENCES**

1. Conroy RM, Pyorala K, Fitzgerald AP, Sans S, Menotti A, et al. (2003) Estimation of ten-year risk of fatal cardiovascular disease in Europe: the SCORE project. Eur Heart J 24: 987-1003.

2. O'Hara GA, Duncan CJ, Ewer KJ, Collins KA, Elias SC, et al. (2012) Clinical assessment of a recombinant simian adenovirus ChAd63: a potent new vaccine vector. J Infect Dis 205: 772-781.

3. Sheehy SH, Duncan CJ, Elias SC, Choudhary P, Biswas S, et al. (2012) ChAd63-MVA-vectored blood-stage malaria vaccines targeting MSP1 and AMA1: assessment of efficacy against mosquito bite challenge in humans. Mol Ther 20: 2355-2368.

4. Douglas AD, Edwards NJ, Duncan CJ, Thompson FM, Sheehy SH, et al. (2013) Comparison of Modeling Methods to Determine Liver-to-blood Inocula and Parasite Multiplication Rates During Controlled Human Malaria Infection. J Infect Dis 208: 340-345.

5. Crosnier C, Bustamante LY, Bartholdson SJ, Bei AK, Theron M, et al. (2011) Basigin is a receptor essential for erythrocyte invasion by Plasmodium falciparum. Nature 480: 534-537.

6. Douglas AD, Williams AR, Illingworth JJ, Kamuyu G, Biswas S, et al. (2011) The blood-stage malaria antigen PfRH5 is susceptible to vaccine-inducible cross-strain neutralizing antibody. Nat Commun 2: 601.

7. Voss S, Skerra A (1997) Mutagenesis of a flexible loop in streptavidin leads to higher affinity for the Strep-tag II peptide and improved performance in recombinant protein purification. Protein Eng 10: 975-982.

8. Miura K, Orcutt AC, Muratova OV, Miller LH, Saul A, et al. (2008) Development and characterization of a standardized ELISA including a reference serum on each plate to detect antibodies induced by experimental malaria vaccines. Vaccine 26: 193-200.

9. Epstein JE, Tewari K, Lyke KE, Sim BK, Billingsley PF, et al. (2011) Live attenuated malaria vaccine designed to protect through hepatic CD8(+) T cell immunity. Science 334: 475-480.

10. Rickman LS, Gordon DM, Wistar R, Jr., Krzych U, Gross M, et al. (1991) Use of adjuvant containing mycobacterial cell-wall skeleton, monophosphoryl lipid A, and squalane in malaria circumsporozoite protein vaccine. Lancet 337: 998-1001.

11. Sedegah M, Kim Y, Ganeshan H, Huang J, Belmonte M, et al. (2013) Identification of minimal human MHC-restricted CD8+ T-cell epitopes within the Plasmodium falciparum circumsporozoite protein (CSP). Malar J 12: 185.

12. Ewer KJ, O'Hara GA, Duncan CJ, Collins KA, Sheehy SH, et al. (2013) Protective CD8(+) T-cell immunity to human malaria induced by chimpanzee adenovirus-MVA immunisation. Nat Commun 4: 2836.

13. Wizel B, Houghten RA, Parker KC, Coligan JE, Church P, et al. (1995) Irradiated sporozoite vaccine induces HLA-B8-restricted cytotoxic T lymphocyte responses against two overlapping epitopes of the Plasmodium falciparum sporozoite surface protein 2. J Exp Med 182: 1435-1445.

14. Gilbert SC, Plebanski M, Harris SJ, Allsopp CE, Thomas R, et al. (1997) A protein particle vaccine containing multiple malaria epitopes. Nat Biotechnol 15: 1280-1284.
